# Transcribed enhancer sequences are required for maize *p1* paramutation

**DOI:** 10.1093/genetics/iyad178

**Published:** 2024-01-03

**Authors:** Lyudmila V Sidorenko, Vicki L Chandler, Xiujuan Wang, Thomas Peterson

**Affiliations:** Department of Plant Sciences, The University of Arizona, Tucson, AZ 85721, USA; Corteva Agriscience, 7300 NW 62nd Ave, Johnston, IA 50131, USA; Department of Plant Sciences, The University of Arizona, Tucson, AZ 85721, USA; Minerva University, 14 Mint Plaza, Suite 300, San Francisco, CA 94103, USA; Corteva Agriscience, 7300 NW 62nd Ave, Johnston, IA 50131, USA; Department of Genetics, Development, and Cellular Biology, Department of Agronomy, Iowa State University, Ames, IA 50010, USA; Department of Genetics, Development, and Cellular Biology, Department of Agronomy, Iowa State University, Ames, IA 50010, USA

**Keywords:** maize *p1* gene, transcriptional silencing, paramutation, DNA methylation, transcription, enhancer, direct repeats, transgene, small RNA

## Abstract

Paramutation is a transfer of heritable silencing states between interacting endogenous alleles or between endogenous alleles and homologous transgenes. Prior results demonstrated that paramutation occurs at the *P1-rr* (*r*ed pericarp and *r*ed cob) allele of the maize *p1* (*pericarp color 1*) gene when exposed to a transgene containing a 1.2-kb enhancer fragment (*P1.2*) of *P1-rr*. The paramutable *P1-rr* allele undergoes transcriptional silencing resulting in a paramutant light-pigmented *P1-rr′* state. To define more precisely the sequences required to elicit paramutation, the *P1.2* fragment was further subdivided, and the fragments transformed into maize plants and crossed with *P1-rr.* Analysis of the progeny plants showed that the sequences required for paramutation are located within a ∼600-bp segment of *P1.2* and that this segment overlaps with a previously identified enhancer that is present in 4 direct repeats in *P1-rr.* The paramutagenic segment is transcribed in both the expressed *P1-rr* and the silenced *P1-rr′.* Transcription is sensitive to α-amanitin, indicating that RNA polymerase II mediates most of the transcription of this sequence. Although transcription within the paramutagenic sequence was similar in all tested genotypes, small RNAs were more abundant in the silenced *P1-rr′* epiallele relative to the expressed *P1-rr* allele. In agreement with prior results indicating the association of RNA-mediated DNA methylation in *p1* paramutation, DNA blot analyses detected increased cytosine methylation of the paramutant *P1-rr′* sequences homologous to the transgenic *P1.2* subfragments. Together these results demonstrate that the *P1-rr* enhancer repeats mediate *p1* paramutation.

## Teaser

Paramutation is an interaction between alleles resulting in heritable silencing of gene expression. First discovered in maize by R. A. Brink in the 1950's, paramutation occurs in both plants and animals; however, understanding what features specify paramutagenic interactions between alleles and/or transgenes remains limited. Here, Sidorenko et al. used transgenic analyses of the maize *pericarp color 1 (p1)* gene to show that a unique ~600 bp DNA sequence arranged as direct repeats overlapping with a transcribed enhancer mediates all aspects of paramutation.

## Introduction

Paramutation is defined as an interaction between homologous alleles that leads to a change in expression of one of the interacting alleles. Paramutation is an epigenetic phenomenon that correlates with reduced transcription (Patterson et al. 1993), increased DNA methylation (Walker 1998; Sidorenko and Peterson 2001; Haring et al. 2010), and a more compact chromatin structure at specific sequences of the paramutated alleles (van Blokland et al. 1997; Stam, Belele, Dorweiler, et al. 2002; Stam, Belele, Ramakrishna, et al. 2002; Louwers et al. 2009). In maize, 5 cases of paramutation have been described; 4 cases involve transcription factors regulating the flavonoid biosynthetic pathway: *red1* (*r1*) (Brink 1956), *booster1* (*b1*) (Coe 1966), *plant color1* (*pl1*) (Hollick et al. 2000), and *pericarp color1* (*p1*) (Sidorenko and Peterson 2001), while an additional case involves the *low phytic acid1* (*lpa1*) locus (Pilu et al. 2009) of the phytic acid biosynthetic pathway. Paramutation-like behaviors have been described for other endogenous and transgenic loci in various plant species (see for review Chandler 2007; Onate-Sanchez and Vicente-Carbajosa 2008; Hollick 2017), including, *Arabidopsis* endogenous genes and transgenes (Luff et al. 1999; Mittelsten Scheid et al. 2003; Xue et al. 2012; Gao and Zhao 2013; Zheng et al. 2015; Bente et al. 2021), petunia and tomato endogenous genes (Hagemann 1969; van Houwelingen et al. 1999; Gouil et al. 2016; Gouil and Baulcombe 2018), and a tobacco transgene (Khaitová et al. 2011). Paramutation-like phenomena have also been reported in animals (Cuzin et al. 2008; Capovilla et al. 2017; Hollick 2017; Dorador et al. 2022).

The *p1* gene encodes a *myb-*like transcription factor that regulates the biosynthesis of flavonoid-derived pigments (phlobaphenes) in mature tissues of maize (Lechelt et al. 1989; Grotewold et al. 1994). Accumulation of phlobaphene pigments in maize ears serves as a convenient indicator of *p1* expression. Alleles of the *p1* gene are designated by a 2-letter suffix with the first letter denoting pigmentation of the kernel pericarp (protective outer layer derived from the ovary wall) and the second letter indicating cob glume pigmentation. For example, alleles with *r*ed pericarp and red cob are denoted as *P1-rr*, alleles with red pericarp and *w*hite cob are referred to as *P1-rw*, alleles with *w*hite pericarp and *r*ed cob are named *P1-wr*, and alleles with *w*hite pericarp and *w*hite cob are indicated as *p1-ww*.

**Fig. 1.**
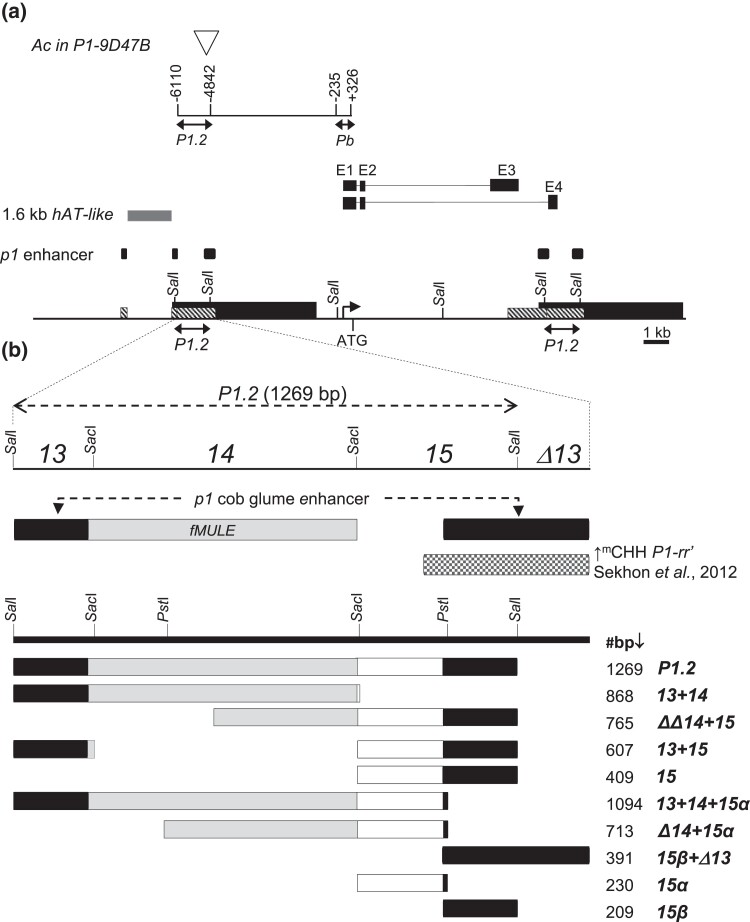
Structure of the *P1-rr* locus and transgenic *P1.2* subfragments. a) The *P1-rr* gene map. Starting at the top, the location of *Ac* transposon insertion that disrupts *P1.2* distal enhancer and results in reduced pericarp pigmentation (Sidorenko et al. 2000) is shown by a triangle. Coordinates of the *P1.2* and *Pb* fragments (double-headed arrows) are shown relatively *P1-rr* transcription start site. The major *P1-rr* transcript containing exons 1, 2, and 3 (E1, E2, and E3) and an alternative *P1-rr* transcript containing exons 1, 2, and 4 (E1, E2, and E4) are indicated by black boxes with introns shown as thin lines. A 1.6-kb *hAT*-like transposable element that disrupts the upstream-most 1.2-kb repeat is indicated by a medium gray box. Copies of the *p1* cob glume enhancer (Zhang and Peterson 2005) are shown as black boxes. Along the *P1-rr* map, the 5.2-kb-long direct repeats (black rectangles) overlapping with smaller 1.2-kb direct repeats (hatched boxes), and *SalI* restriction sites are indicated. Bent arrow shows *P1-rr* transcription start site at position +1; ATG indicates the *P1-rr* translation start site. Below the map: double-headed arrows indicate the location of the *Sal*I-delimited upstream and downstream copies of the *P1.2* fragment, which are 99.84% identical (Su et al. 2020). b) Schematic drawings of the transgenic *P1.2* subfragments. Locations of the major *P1.2* subfragments and *Sal*I restriction sites are shown on the top of b (Lechelt et al. 1989). The truncated copy of *13* (*Δ13*) lacks the downstream *Sal*I (Supplementary Fig. 1b). Locations of *p1* cob glume enhancer (Zhang and Peterson 2005) are shown as black rectangles. A fractured *MULE* (*fMULE*) is shown as a light gray rectangle (Goettel and Messing 2010; Goettel and Messing 2013a). The fragment of *P1-rr′* with increased CHH methylation reported by Sekhon et al. 2012 is shown as checkered box. The *Sal*I, *Sac*I, and *Pst*I restriction sites are indicated along the thick black line. The *P1.2* subfragments are diagrammed as rectangles below the restriction map. Within each construct diagram, black rectangles indicate sequence of the *p1* cob glume enhancer, light gray rectangles diagram *fMULE*, and open rectangles show sequence unique to *p1* locus. For simplicity, the basal *P1-rr* promoter and 5′UTR that were included in all transgene constructs (Materials and methods) are not shown. Construct names are indicated to the right of the construct diagrams. Length of the *P1.2* subfragments are indicated in base pairs (bp) before each construct name. Detailed sequence information for the *P1.2* subfragments is shown in Supplementary Fig. 1a.

The *P1-rr* allele has a complex structure with the coding sequence flanked by compound direct repeats (Fig. 1a). Two long direct repeats of 5.2 kb (black rectangles) are located immediately upstream and downstream of the *P1-rr* coding sequence and are juxtaposed with 1.2-kb direct repeats (hatched rectangles) (Fig. 1a). The upstream-most 1.2-kb repeat is truncated and interrupted by a 1.6-kb *hAT*-family transposon (Goettel and Messing 2010), while the downstream 1.2-kb repeats overlap with the exons 3 and 4 of *P1-rr* (Grotewold et al. 1991; Goettel and Messing 2010). A fractured *Mu-like* transposable element (*fMULE*; Fig. 1b) is present within 1.2-kb repeats, 1 copy within the upstream 1.2-kb repeats and 2 copies in the downstream 1.2-kb repeats (Goettel and Messing 2013a). The 1.2-kb direct repeats contain *Sal*I restriction sites (Lechelt et al. 1989) that delimit the upstream *P1.2* (located ∼5-kb upstream of *P1-rr* transcription start site, at −6,110 to −4,842 in AF209212.1) and the downstream *P1.2* (overlapping with the alternatively spliced *p1* transcript; Fig. 1a). Insertion of *Ac* transposon into the upstream copy of the *P1.2* sequence (Fig. 1a) of *P1-rr* results in reduced ear pigmentation, indicating the importance of this upstream element for *p1* expression (Athma et al. 1992; Moreno et al. 1992; Sidorenko et al. 2000). Subsequent transient and stable transgenic plant assays confirmed enhancer function of the *P1.2* fragment (Sidorenko et al. 1999, 2000). Comparing phenotypes, RNA expression patterns, and the upstream regulatory sequences of *P1-rr* and *P1-rw* alleles identified a 386-bp sequence within the upstream-most 1.2-kb repeat of *P1-rr* as a cob glume enhancer (Fig. 1a and b) (Zhang and Peterson 2005, 2006). Together, these studies of structural variation of endogenous alleles and functional tests in transgenic experiments demonstrated the importance of the *P1.2* enhancer fragment in *P1-rr* regulation.

Paramutation at the *p1* gene was first observed in experiments that studied epigenetic interactions between the endogenous *P1-rr* allele and transgenes carrying fragments of *P1-rr* regulatory region (Sidorenko and Peterson 2001). When introduced as a transgene, the *P1.2* enhancer, fused to the basal *P1-rr* promoter (*Pb*) and *GUS* gene (*P1.2b::GUS*), caused silencing of the endogenous *P1-rr* allele, resulting in reduced pigmentation and variable patterning of pericarp and cob glumes. Additional tests demonstrated that *Pb* was not required and that *P1.2* was sufficient for paramutation (Sidorenko and Chandler 2008). By convention with paramutation nomenclature, silenced *P1-rr* epialleles are termed *P1-rr′,* with the prime mark signifying the epigenetically silenced state resulting from paramutation. The silenced *P1-rr′* state was heritable and caused secondary silencing (secondary paramutation or paramutagenicity) of a naïve (no previous exposure to a transgene) *P1-rr* allele in the absence of the inducing *P1.2* transgene (Sidorenko and Peterson 2001; Sidorenko and Chandler 2008). Molecular analyses revealed that the transgene-induced silenced state was associated with increased DNA methylation of the endogenous 1.2-kb repeats and reduced *P1-rr* transcription (Sidorenko and Peterson 2001; Sidorenko and Chandler 2008). The frequency of silencing induced by the *P1.2*-containing transgenes varied between independent transgenic events, as did the heritability and secondary paramutagenicity of the newly induced *P1-rr′* states (Sidorenko and Peterson 2001; Sidorenko and Chandler 2008). Generally, progeny derived from strongly silenced *P1-rr′* epialleles were more likely to remain heritably silenced and to exhibit secondary paramutation than progeny from weakly silenced *P1-rr′* alleles, which frequently reverted back to the fully pigmented red *P1-rr* state in the absence of the inducing transgene (Sidorenko and Peterson 2001). These observations indicated that newly established *P1-rr′* silencing was metastable, although stable lineages could be selected. This is similar to the properties of alleles participating in paramutation at *pl1* and *r1* loci where metastable states have been reported (Styles and Brink 1966; Brink et al. 1968; Styles et al. 1973; Hollick et al. 1995) but distinct from *b1* paramutation in which silencing and secondary paramutation are extremely stable (Coe 1959, 1966).

In addition to transgene-induced paramutation of *P1-rr* (Sidorenko and Peterson 2001; Sidorenko and Chandler 2008), spontaneous epialleles of *P1-rr* with patterned pericarp and red cob designated as *P1-pr* have been described. The 2 independently isolated *P1-pr* epialleles (Das and Messing 1994; Sekhon et al. 2012) are phenotypically indistinguishable from each other and from *P1-rr′* induced by the *P1.2* transgene, but they differ in the stability of the silenced states and their paramutation characteristics. Similar to *P1-rr′* (Sidorenko and Peterson 2001), the *P1-pr* isolate characterized by Das and Messing (1994) is mostly stable, although low-frequency reversions were reported (Das and Messing 1994; Goettel and Messing 2013a). In contrast, the *P1-pr^TP^* isolate is very stable with no revertants observed in ∼1,000 plants examined (Sekhon et al. 2012). Interestingly, the *P1-pr* isolated by Das and Messing is highly paramutagenic (Goettel and Messing 2013b), while *P1-pr^TP^* is not paramutagenic and behaves as a recessive allele in crosses with *P1-rr* (Sekhon et al. 2012). Cytosine methylation assays showed that *P1-rr′* and both *P1-pr* isolates have similar high densities of symmetric CG and CHG (H is any base but G) methylation in the region of the 1.2-kb fragment. However, there is a distinct difference in nonsymmetric CHH methylation; the paramutagenic *P1-rr′* and *P1-pr* had ∼10% and ∼5% CHH methylation, respectively (Sekhon et al. 2012; Goettel and Messing 2013a), while no detectable CHH methylation was observed for nonparamutagenic *P1-pr^TP^* (Sekhon et al. 2012). Because CHH methylation depends on presence of small RNAs (sRNAs) involved in RNA-dependent DNA methylation (RdDM) (Matzke and Mosher 2014), the difference in nonsymmetric CHH methylation was proposed to be a distinguishing molecular mark between the paramutagenic and nonparamutagenic *P1-rr* epialleles (Sekhon et al. 2012).

Further insights into the molecular mechanisms involved in *p1* paramutation were obtained in genetic experiments with RdDM mutants that affect paramutation at other maize loci. The first gene shown to be required for *p1* paramutation was *mop1* (*mediator of paramutation 1*) (Dorweiler et al. 2000; Alleman et al. 2006). The *Mop1* gene is allelic to *Rmr6* (*required to maintain repression 6*) (Erhard et al. 2009) and encodes an RNA-dependent RNA (RDR) polymerase orthologous to *Arabidopsis* RDR2 (Alleman et al. 2006). The *mop1-1* mutation disrupted the establishment of *p1* paramutation, but in contrast to *b1* paramutation in which silencing was reversed in the first generation (Dorweiler et al. 2000), effects on the maintenance of *P1-rr′* silencing were delayed; several generations of exposure to the homozygous *mop1-1* mutation were required to restore pigmentation of *P1-rr′* (Sidorenko and Chandler 2008). Slow reactivation of *P1-rr′* in *mop1* mutant background correlated with modest increase in *P1-rr* transcript levels (∼40%), slight decrease of repressive H3K9me^2^ histone mark (∼30%), and reduction of CHH DNA methylation from 7.1 to 3.8%, while CG and GHG methylation was not significantly different (Wang et al. 2017). The second gene shown to be required for *p1* paramutation is *mop2* (Sidorenko et al. 2009). This gene is allelic to *Rmr7* (Stonaker et al. 2009) and encodes one of 3 paralogs of the second largest subunit of RNA polymerases IV and V [Pol-IV/V; also referred to as NRP(D/E)2a (Haag et al. 2014)]. In the heterozygous state, the *Mop2-1* mutation disrupted the establishment of *pl* paramutation, and this dominant mutant behavior was similar to the results with *b1* paramutation (Sidorenko et al. 2009). However, in contrast to *b1* paramutation, the *Mop2-1* allele did not affect maintenance of *p1* paramutation (Sidorenko et al. 2009). The effects of mutations in *mop1* and *mop2* genes that are orthologous to genes in the *Arabidopsis* RdDM pathway demonstrates the involvement of RNA-mediated regulation in *p1* paramutation (Sidorenko and Chandler 2008; Sidorenko et al. 2009). In contrast to the *mop1-1* and *Mop2-1* mutations, the dominant *Ufo1-1* (*Unstable factor for orange1*) mutation (Wittmeyer et al. 2018) disrupts previously established *P1-rr′* silencing in the first generation of exposure to the mutation (Sekhon et al. 2012) suggesting a critical requirement for UFO1 in maintaining *P1-rr′* silencing. At the molecular level, *Ufo1-1* mutation arose via a *CACTA* transposon insertion and increased expression of a *Poaceae*-specific protein with no annotated function (Wittmeyer et al. 2018). While the mechanism by which *Ufo1-1* derepresses silenced *P1-rr′* is unknown, the presence of *Ufo1-1* resulted in decreased cytosine methylation within a 443-bp subfragment of the *P1.2* enhancer in all sequence contexts (CG, CHG, and CHH) and reduced accumulation of the repressive H3 lysine 9 di-methylated histone modification (H3K9me2) (Sekhon et al. 2012). Together, these genetic studies show that, similar to other cases of maize paramutation (Dorweiler et al. 2000; Alleman et al. 2006; Erhard et al. 2009; Stonaker et al. 2009), establishment of *p1* paramutation depends on RNA-mediated silencing mechanisms, while maintenance of *P1-rr′* silencing has distinct genetic requirements that would benefit from additional studies. These additional studies may include factors that have been shown to regulate *pl1* and *b1* paramutations, including but not limited to PICKLE-like chromodomain DNA-binding 3 (CHD3) protein that is required to maintain *pl1* paramutation but is dispensable for maintenance of *b1* paramutation (Deans et al. 2020) and AGO104 that disrupts *b1* paramutation (Aubert et al. 2022).

The aim of the present study is to further define the minimal sequences within the *P1.2* fragment required for paramutation. We designed a series of *P::GUS* constructs containing subfragments of *P1.2* (Fig. 1b) and stably transformed these constructs into maize. The resulting transgenes were tested for their ability to induce silencing of *P1-rr*, and the heritability and secondary paramutagenicity of the resulting *P1-rr′* states were characterized. These experiments identified a minimal paramutagenic sequence of ∼600 bp within *P1.2* and further documented the involvement of cytosine methylation, transcription, and sRNA accumulation in paramutagenic silencing.

## Materials and methods

### Plasmid construction

Constructs used in the study were assembled using standard molecular cloning techniques in a vector described in Sidorenko et al. (1999). All constructs contained the *Pb* fragment (−235 to +326, AF209212.1) including the basal *P1-rr* promoter and 5′UTR of the *P1-rr* gene, which were fused to the maize *AdhI* gene intron I, the *β-glucuronidase* (*GUS*) reporter gene, and the potato *Proteinase Inhibitor II* (*PinII*) terminator. A diagram of the *P1.2* subfragments used in this study is shown in Fig. 1a and b with detailed information in Supplementary Fig. 1. The *P1.2* subfragments *13*, *14*, and *15* are the same as *P1-rr* locus fragments 13, 14, and 15 described in Lechelt et al (1989). The *P1.2b::GUS* and *Pb::GUS* constructs were previously described (Sidorenko and Chandler 2008). For simplicity, in this study, *P1.2b::GUS* is referred to as *P1.2*, and *Pb::GUS* is referred to as *Pb*.

### Plant transformation

Transgenic plants production was essentially as in Sidorenko and Chandler (2008). Briefly, transgenic maize plants were produced at the Iowa State University Transformation Facility (ISU PTF) using a biolistic bombardment protocol. Plasmids of interest and the pBAR184(−) selectable construct were cobombarded into *Hi-II* immature embryos (Armstrong 1994; Frame et al. 2000). *Hi-II* carries the *p1-ww* allele that does not participate in paramutation. The T_0_ transgenic plants were crossed with a *P1-rr* tester (see description of genetic stocks) to produce seeds for further testing (Supplementary Fig. 2).

### Molecular analyses of transgenic callus and maize transgenic plants

Transgenic callus clones were screened for the presence of transgenic constructs of interest using DNA blot analyses as described by Sidorenko and Chandler (2008). Briefly, genomic DNA was digested with restriction enzymes, electrophoresed, blotted, and hybridized with a ^32^P-labeled 788-bp *Nco*I-*Alw*NI fragment from the *GUS* gene coding region. Only *GUS*-positive callus clones were regenerated. Transgenic lines were evaluated in T_0_ and/or T_1_ generations as follows: leaf genomic DNA was digested with restriction enzymes that released the entire *P::GUS* cassette; digested DNA was electrophoresed, blotted, and hybridized with ^32^P-labeled GUS and/or 15 probes; and the number of discrete bands were counted and reported in Supplementary Fig. 3. Additionally, transgene copy number was estimated by comparison of DNA gel blot hybridization signals of transgene and endogenous *P1-rr* bands with probe 15 using Phoretix 1D software package (Sigma-Aldrich). For this, bands were detected as recommended by software developer and background subtracted. Lanes containing samples from nontransgenic plants were used as controls to estimate probe 15 copy number in lanes containing transgenic samples. Endogenous probe 15 copies were subtracted from the total estimated probe 15 copy number, and the resulting estimated probe 15 copy numbers are reported in Supplementary Fig. 3.

### Genetic stocks

The *P1-rr* stock used in this study was the standard *P1-rr4B2* allele (Grotewold et al. 1991). Except for initial crosses with T_0_ transgenic plants that were conducted at the ISU PTF, large-scale genetic experiments with transgenic plants employed isolation fields where transgenic families were detasseled and their ears were allowed to open pollinate with nontransgenic testers planted in alternating rows. Because isolation fields were in Arizona, we developed hybrid *P1-rr* and *p1-ww* tester stocks that performed well in the dry and hot weather. For the hybrid *P1-rr* stock, we crossed the near isogenic inbred lines of *P1-rr4B2[4Co63]* and *P1-rr4B2[W23]*. For the hybrid *p1-ww* stock, we crossed *p1-ww[4Co63]* and *p1-ww[A619]* inbred lines. The *p1-ww[4Co63]* allele is neutral to paramutation and carries a single copy of fragment 15 (Sidorenko and Peterson 2001; Sidorenko and Chandler 2008; Goettel and Messing 2013b). Similar to *p1-ww[4Co63]*, the null *p1-ww[A619]* allele carries a single copy of fragment 15 (Szalma et al. 2005; Morohashi et al. 2012) and was not observed to participate in paramutation (Sidorenko, unpublished).

### Genetic tests with transgenic plants

Diagrams of crosses used in this study are shown in Supplementary Figs. 2, 4, and 6. In these diagrams, presence of a transgene is indicated as *TR*, and asterisk is used to indicate *P1-rr* that was exposed to the transgene. To identify transgenic plants in segregating families, all plants were screened for tolerance to Ignite herbicide (Bayer) using the leaf painting assay. For this, a permanent black marker was used to mark the location of herbicide application on a fully expanded maize leaf near the leaf tip. Herbicide solution (1% v/v) was applied with a sponge next to the marker line. Three days after application, herbicide tolerance was visually scored; plants with leaf damage were scored as sensitive, while plants with no damage as tolerant. To confirm the absence of transgenes in the herbicide-sensitive plants, DNA from these plants was subjected to PCR amplification with GUS primers (forward 5′-GCGTCGGCATCCGGTCAGTGGC-3′ and reverse 5′-TCTGCCGTTTCCAAATCGCCGC-3′) and/or DNA slot blot analyses with a ^32^P-labeled *Nco*I-*Alw*NI GUS fragment probe. To distinguish *P1-rr/P1-rr* from *P1-rr/p1-ww* genotypes in the secondary paramutagenicity tests, DNA of all nontransgenic plants were subjected to PCR amplification and genotyping by sequencing. The sequences of the primers used for genotyping were obtained from R. Sekhon (forward 5′-GTCGCGTGGGTCTTCGTTCAG-3′ and reverse 3′-ATGCAAGTGACAAATTATCATGATGGCA-5′). Resulting 452-bp PCR product spanned a simple sequence repeat polymorphism­­ (Supplementary Fig. 1a) that was used for genotyping. In this assay, the *P1-rr/P1-rr* plants produced uninterrupted sequence traces, while *P1-rr/p1-ww* plants displayed easily identifiable shift in sequencing traces after the polymorphism (GTGTGT in *P1-rr* and GTGTGTGT in *p1-ww*; Supplementary Fig. 1a). PCR products were sequenced at the University of Arizona Genetics Core Facility using the reverse primer described above.

### Statistical analysis

To compare frequency of silenced ears of each construct with that of the control *P1.2* construct, we carried out 2 sample tests for equality of proportions with Yates’ correction for continuity. The test hypotheses to compare frequency of silenced ears are as follows: H_0_, The frequency of silenced ears of a construct with a fragment of *P1.2* is equal to frequency of silenced ears of the construct with complete *P1.2*; H_A_, The frequency of silenced ears of a construct with a *P1.2* fragment is not equal to frequency of silenced ears of the construct with complete *P1.2*. The *P*-values obtained from the hypothesis testing were adjusted using Bonferroni–Holm method for multiple comparison correction.

### DNA blot analyses of cytosine methylation

DNA blot analyses were conducted using leaf genomic DNA extracted by a CTAB protocol (Saghai-Maroof et al. 1984; Wise and Schnable 1994). Restriction enzymes not sensitive to cytosine methylation (*Eco*RI and *Kpn*I) were used to assess the transgene locus structure, while enzymes sensitive to CpG (*Sal*I) and CpNpG (*Pst*I) cytosine methylation were used to assess methylation of the endogenous *P1-rr* allele. Standard DNA blot protocol (Sidorenko et al. 2000; Sidorenko and Peterson 2001) involving DNA digestion, electrophoresis, transfer to Nylon membrane, hybridization with ^32^P random labeled probe, washing, and image acquisition was used in this study.

### Nuclear run-on analysis

Nuclear run-on analysis was conducted using fresh tissues harvested from field grown plants. Young inner leaf sheaths were collected from plants at the V7 growth stage. Young inner husks were collected from developing ears before silk extrusion. Immature pericarps were collected from kernels 20 days after pollination. To extract nuclei, 5 g of a respective tissue was ground in liquid nitrogen and resuspended in 30 ml of ice-cold extraction buffer (Sidorenko and Chandler 2008). The slurry was filtered through Miracloth (Calbiochem) and 53 µm nylon mesh. The filtrate was transferred to centrifuge tubes and centrifuged for 15 min at −10° C and 6,000 *g*. The supernatant was removed, and nuclei were resuspended using a soft paint brush, washed with 20 ml of the wash buffer, pelleted again, and resuspended in 2 ml of the wash buffer (Lisch et al. 2002; Carey et al. 2004). The remaining run-on reaction was as described by Dorweiler et al. (2000). Total RNA from nuclei was extracted using TRIzol reagent (Life Technologies) according to the manufacturer recommendations. Hybridization was as described previously (Dorweiler et al. 2000). To prepare RNA probes for the *13*, *15α*, and *15β* subfragments and the 18S control, PCR-amplified fragments containing the T3 promoter were used as templates. The in vitro transcription reactions were performed using T3 RNA polymerase as recommended by the manufacturer (New England BioLabs). The maize *Ubiquitin2* (*Ubi2*) RNA probe was prepared using SP6 RNA Polymerase and the linearized pCA210 plasmid (Christensen et al. 1992). A *DNA*seI digestion was used to remove DNA template from the in vitro synthesized RNA probe preparations. To prepare slot blots, 200 ng of denatured RNA was immobilized on GeneScreenPlus Nylon membrane (PerkinElmer). After hybridization and washing, blots were scanned using a BioRad Pharos scanner, and pixel volumes were determined using the accompanying software package. After subtracting the background signal, pixel volumes per cm^2^ were normalized to the pixel volumes obtained for *Ubi2*. In experiments with α-amanitin, pixel volumes were not normalized as transcription of all genes assayed in this experiment was expected to be affected.

### sRNA blots

Three grams of young ears (3–5 cm long) were used to extract sRNAs as described by Sidorenko *et al.* (2009). Briefly, total RNA was isolated using TRIzol reagent (Life Technologies). Larger RNAs were depleted by precipitation with 50% polyethylene glycol MW 8000 and centrifugation. The aqueous phase containing sRNAs was extracted with phenol:chlorophorm:isoamyl-alcohol (24:1:1), precipitated with 1/10 volume of 3 M NaOAC and 2.5 volume of 100% ethanol, and pellets dissolved in RNAse-free water. Equal amounts (100 µg) of enriched sRNA fraction were loaded in each lane of the RNA blots. Electrophoresed RNA was blotted onto GeneScreenPlus Nylon membrane (PerkinElmer) and hybridized with ^32^P 5′-end-labeled DNA/locked nucleic acid (LNA) oligos synthesized by Sigma-Proligo. The sequence of the DNA/LNA oligos is shown below with the LNA-modified bases preceded by a plus (+) sign. The forward oligo (ACC + AATCG + CCGCT + GCAGC + AGTGC + CCAGT + GAGTG + GTGCCA + CCACGC) recognized the antisense strand (relative to the *p1* coding sequence). The reverse oligo (GCG + TGGTG + GCACC + ACTCA + CTGGG + CACTG + CTGCA + GCGGC + GATTG + GT) recognized the sense strand (relative to the *p1* coding sequence). Location of the DNA/LNA probes is shown in Supplementary Fig. 1a. MicroRNA Marker (NEB #N2102) was used for estimation of sRNA sizes.

## Results

### Transgenes carrying *P1.2* subfragments had complex multicopy transgene insertions

Transgenic plants were produced using a biolistic bombardment protocol in which *P::GUS* constructs were cobombarded with the pBAR184(−) selectable construct (Armstrong 1994; Frame et al. 2000). As expected for biolistic cotransformation method, transgenic events exhibited multicopy *P::GUS* transgene insertions with a wide variation of copy numbers (2 to 163 copies; Supplementary Fig. 3). A combination of herbicide leaf paint test and molecular analyses were used to evaluate transgene segregation in T_1_ plants (Materials and methods). Results revealed that roughly 1/3 of the transgenic events (24 out of 77) exhibited deviations from the expected 1 herbicide-tolerant:1 herbicide-sensitive segregation (Supplementary Fig. 3). Among events with skewed segregation, 13 events had higher than expected and 11 events lower than expected frequencies of herbicide-tolerant plants. Higher than expected frequency of herbicide-tolerant plant could be a result of multiple unlinked transgene insertions (Spencer et al. 1990; Register et al. 1994), while lower than expected frequency of herbicide-tolerant plants could occur when the selectable marker becomes silenced (Kumpatla et al. 1997; Rajeevkumar et al. 2015). While we were not able to follow up on every case of deviation from the expected 1:1 segregation, we identified 7 cases of unlinked transgene insertions that increased number of HT plants (indicated by red stars in Supplementary Fig. 3). We also identified 93 plants sensitive to herbicide and carrying *P::GUS* transgenes distributed among 24 transgenic events (Supplementary Fig. 3), indicating that silencing of selectable marker occurred sporadically in many transgenic events.

Because the relationship between transgenic event structure and its ability to induce *P1-rr* silencing is of interest, we explored correlation analyses using data in Supplementary Fig. 3. We detected positive correlation between number of transgenic bands and *P1-rr* silencing for *14 + 15a* and *Pb* constructs (*R*^2^ 0.57, *P* = 0.004 and *R*^2^ 0.65, *P* = 0.05, respectively), while the other constructs showed no significant correlation (not shown). Using estimated copy number for correlation analyses did not reveal significant correlation for any of the constructs (not shown). These results indicate that the highly variable and rearranged transgene structures generated by biolistic bombardment are not suitable for evaluation of copy number impact on *P1-rr* silencing.

In conclusion, our results of transgenic events characterization are similar to the previously published results reporting biolistic bombardment to produce complex, multicopy transgene insertions that can exhibit sporadic selectable marker silencing. Despite these limitations, transgenic plants served as the essential enabling tool in functional dissection of sequences within the required for *p1* paramutation *P1.2* fragment.

### Sequences within subfragment *15* of the *P1.2* enhancer are required for transgene-induced *P1-rr* silencing

**Fig. 2.**
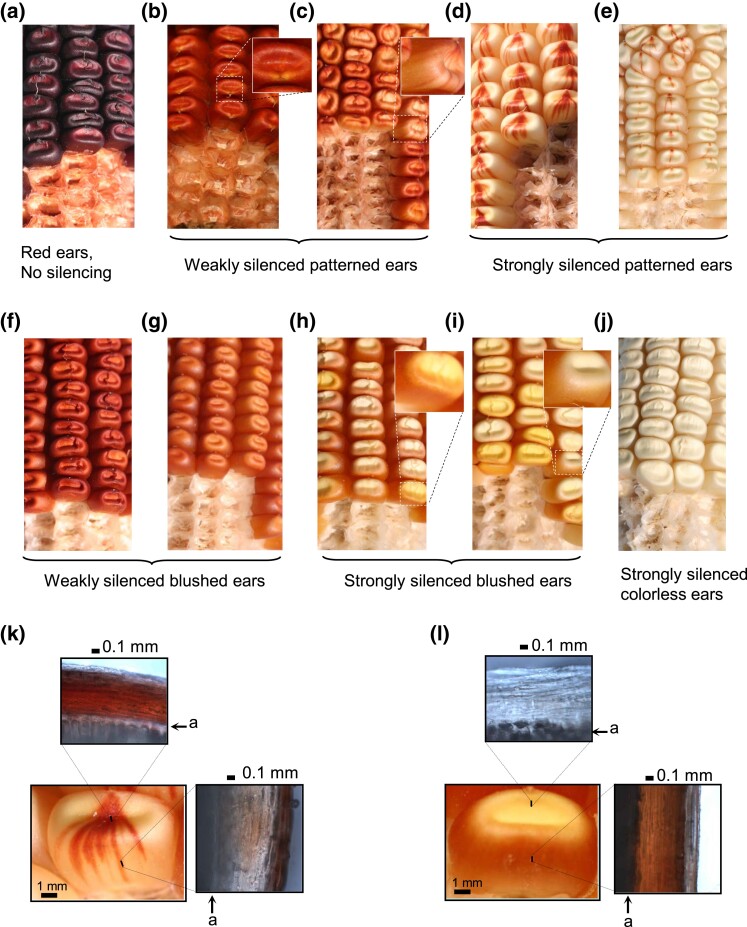
Silencing phenotypes of *P1-rr* induced by the *P1.*2 deletion transgenes. a) The active *P1-rr* allele with dark red pericarp and glumes. Photos b through j) show phenotypes of silenced ears. b and c) Weakly silenced patterned ears with slight reduction of pericarp pigmentation and red cob glumes. Enlargements show slightly darker striping on kernel pericarps. d, e) Strongly silenced patterned ears with pigmentation that varies from heavy striped in d to nearly colorless pericarp with residual pigment at the silk scar in e. The color of cob glumes in these ears varies from pink to near colorless. f and g) Weakly silenced blushed ears have light red to orange pericarp with no visible striping. Please, note that cob glume pigmentation is much lighter than in weakly silenced patterned ears shown in b and c. h, i) Strongly silenced blushed ears have pigmented pericarp gown (sides of a kernel) and lighter pigmented or colorless pericarp crown (top of a kernel). The cob glumes have very little pigment and vary from light pink to colorless. j) The strongest transgene-induced silencing phenotype had colorless ears with no visible pigment in pericarp or cob glumes. k, l) Pigment deposition in representative patterned and blushed kernels, respectively. Photo inserts next to each kernel show thin sections of pericarp prepared with handheld razor blade and examined under binocular microscope. In the patterned kernel, cutting through the pigmented stripe showed that pigment accumulates in the crown and outer cell layer of pericarp gown. In the blushed kernel, pericarp crown is colorless while inner layers of pericarp gown are pigmented. Black bars denote the scale in millimeters (mm). Letter “a” indicates aleurone.

**Fig. 3.**
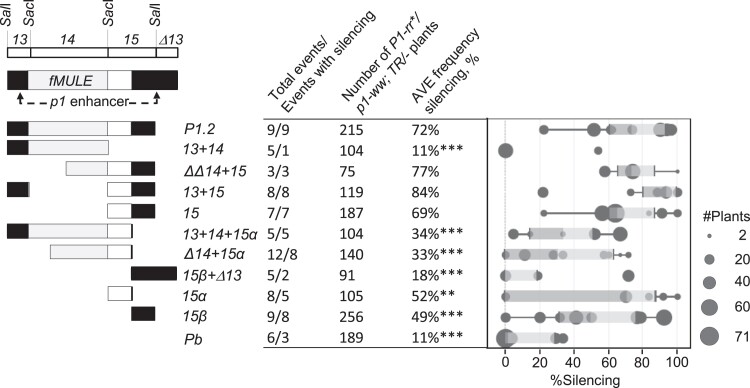
Induction of *P1-rr* silencing by transgenes carrying *P1.2* subfragments. A simplified diagram of *P1.2* subfragments is shown on the left of the figure. Location of *p1* cob glume enhancer, *fMULE*, and major restriction sites is shown on top. Shading within transgenic *P1.2* subfragments is similar to Fig. 2; black rectangles indicate sequence of the *p1* cob glume enhancer, light gray rectangles indicate diagram *fMULE*, and open rectangles show DNA that is unique to *p1* locus. The table in the middle of the figure presents a summary of the number of events, number of plants assayed, and average frequency of *P1-rr* silencing within the informative transgenic p1-ww/P1-rr*, *TR/−* plants (Supplementary Fig. 2). The average frequency of silencing for a construct was calculated as sum of frequencies of silencing for each event divided by the number of tested events (Materials and methods). Statistically significant differences relative the *P1.2* control are indicated by 2 stars for *P* < 0.001 and 3 stars for *P*≤0.0001. *P*-values were calculated by 2-sample test for equality of proportions with Yates’ correction for continuity, followed by Bonferroni–Holm adjustment for multiple comparisons (Materials and methods). The box plot on the right shows silencing frequencies for individual events with each of the dots sized proportionally to the number of transgenic plants assayed for that event. Light- and dark-shaded areas of the box plot indicate 2 middle quartiles with the dividing line indicating median frequency of silencing. The whiskers indicate 1.5 times the minimum and maximum of the respective interquartile range. Additional details regarding transgene segregation, results of molecular analyses, and frequencies of strong and weak *P1-rr* silencing are in Supplementary Fig. 3.

To assay for silencing of *P1-rr*, primary T_0_ transgenic plants homozygous for *p1-ww* and hemizygous for a transgene (*TR/-*) were crossed with the nontransgenic homozygous *P1-rr* tester and progeny plants evaluated for silencing (Materials and methods). In these crosses, exposure of naive *P1-rr* to a transgene is signified by asterisk (Supplementary Fig. 2). As expected, all nontransgenic T_1_  *P1-rr* ears (not shown) had dark red pericarp and cob pigmentation (Fig. 2a). This result is in agreement with prior observations that *P1-rr* is a stable allele and does not undergo appreciable spontaneous silencing (Das and Messing 1994). In contrast, many transgenic plants produced ears with reduced *P1-rr* pigmentation (Fig. 2b–j). The *P1.2* transgene construct, containing the complete 1,269-bp enhancer fragment and used as a positive control for silencing (Sidorenko and Chandler 2008), induced *P1-rr* silencing in all 9 tested events (Fig. 3; Supplementary Fig. 3). The average silencing frequency, calculated as average of silencing frequencies of individual events, was 72%, and the majority of affected plants exhibited phenotypically strong silencing (Supplementary Fig. 3) similar to that shown in Fig. 2d, e, and j.

To further localize sequences required for silencing, 9 constructs containing subfragments of *P1.2* were tested. Subfragments of *P1.2* (Fig. 1b) were developed (Materials and methods) based on previously characterized *P1-rr* restriction fragments 13, 14, and 15 (Lechelt et al. 1989). One of the constructs (*13 + 14*) contained *13* and *14* subfragments and lacked *15*. In contrast to strong and frequent silencing induced by *P1.2*, this construct exhibited only phenotypically weak *P1-rr* silencing (Fig. 2b and c) only in 1 out of 5 tested events, resulting in average frequency of 11% (Fig. 3; Supplementary Fig. 3). Performance of this construct was similar to that of the *Pb* control construct (Sidorenko and Chandler 2008) that contained only the *P1-rr* basal promoter and 5′UTR and exhibited phenotypically weak silencing in 3 out of 6 tested events with average frequency of 11% (Fig. 3; Supplementary Fig. 3). This result showed that sequences within *13* and *14* were not sufficient to induce frequent and phenotypically strong silencing and that subfragment *15*, absent from this construct, is essential for transgene-induced silencing of *P1-rr*. Indeed, the critical role of *15* was confirmed by constructs containing partial or complete fragment *15* sequences (Fig. 3; Supplementary Fig. 3). Three of these constructs (*ΔΔ14 + 15*, *13 + 15*, and *15*) contained the entire fragment *15*; all tested events induced phenotypically strong *P1-rr* silencing (Supplementary Fig. 3), and average frequencies of silencing (Supplementary Fig. 3; 77, 84, and 69%, respectively) were not statistically different from the full-length *P1.2* (72%). Five additional constructs contained one of 2 subfragments of *15* (*15α* or *15β*). For these constructs, not all events induced *P1-rr* silencing, and average silencing frequencies were overall lower (18–52%) than those for the complete *P1.2* control construct (Fig. 3; Supplementary Fig. 3). Together, these results show that the 864 bp of fragments *13* and *14* were not sufficient, while the 409-bp fragment *15* was necessary and sufficient for phenotypically strong and frequent silencing of *P1-rr*.

### Sequences required for high heritability of transgene-induced *P1-rr* silencing are located within the *13* and *15* subfragments

**Fig. 4.**
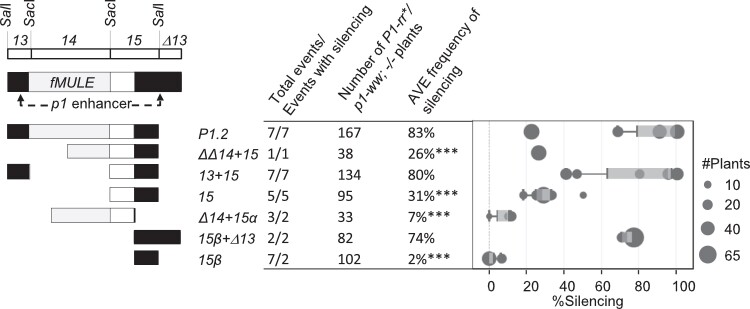
Heritability of *P1-rr* silencing induced by transgenes carrying *P1.2* subfragments. A simplified diagram of *P1.2* subfragments is shown on the left of the figure. Location of *p1* cob glume enhancer, *fMULE*, and major restriction sites is shown on top. Shading within transgenic *P1.2* subfragments is similar to Fig. 1; black rectangles indicate sequence of the *p1* cob glume enhancer, light gray rectangles diagram *fMULE*, and open rectangles show DNA that is unique to *p1* locus. The table in the middle of the figure presents a construct level summary for the informative nontransgenic P1-rr*/P1-ww, −/− plants (Supplementary Fig. 4). Number of events/plants tested and average frequency of silencing (Materials and methods) for each construct are shown. Three stars indicate statistically significant differences (*P* < 0.0001) relative the *P1.2* control. *P*-values were calculated by 2-sample test for equality of proportions with Yates’ correction for continuity, followed by Bonferroni–Holm adjustment for multiple comparisons (Materials and methods). The box plot on the right shows silencing frequencies for individual events with each of the dots sized proportionally to the number of transgenic plants assayed for that event. Light- and dark-shaded areas of the box plot indicate 2 middle quartiles with the dividing line indicating median frequency of silencing. The whiskers indicate 1.5 times the minimum and maximum of the respective interquartile range. Additional information regarding segregation of herbicide tolerance, frequencies of strong, and weak transgene silencing is shown in Supplementary Fig. 5.

Previous studies have shown that silencing of *P1-rr* induced by a *P1.2* transgene remained heritable even after the transgene was removed by genetic segregation (Sidorenko and Peterson 2001; Sidorenko and Chandler 2008). To assay the heritability of the P1-rr*-silenced states induced by subfragments of *P1.2*, transgenic plants were crossed with the nontransgenic *p1-ww* tester (Supplementary Fig. 4). Progeny plants containing P1-rr* and lacking a transgene were identified (Materials and methods). Because previous results with the *P1.2* transgenes demonstrated that the heritability of *P1-rr* silencing was low in progeny of weakly silenced *P1-rr* ears (Sidorenko and Peterson 2001), heritability of P1-rr* silencing was tested only in progeny of strongly silenced ears (similar to those shown in Fig. 2d, e, and h–j). In this way, we tested heritability of transgene-induced *P1-rr* silencing for 7 constructs; the results are shown in Fig. 4 and Supplementary Fig. 5. For the control *P1.2* construct, silencing was heritable in all 7 tested transgenic events; on average 83% of the nontransgenic ears per event were silenced. Similarly, *P1-rr* silencing induced by the *13*-containing constructs, *13 + 15* and *15β + Δ13*, was highly heritable for all tested events; average frequency of silencing per event was 80 and 74%, respectively. In contrast, not all events of constructs lacking *13* (*ΔΔ14 + 15*, *15*, *Δ14 + 15α*, and *15β*) exhibited heritable *P1-rr* silencing. For these constructs, the average frequency of heritable silencing was lower than that for the *P1.2* control (26%, 31%, 7%, and 2%, respectively). Furthermore, the majority of silenced ears (Supplementary Fig. 5) exhibited significant amounts of residual ear pigmentation similar to that in Fig. 2b, c, f, and g. Thus, these results showed that, while not required for establishing silencing of *P1-rr*, the *13* subfragment was required in addition to subfragment *15* to confer high heritability of *P1-rr* silencing in the absence of the inducing transgene.

### Subfragments *13* and *15* contain sequences required for strong secondary paramutation

**Fig. 5.**
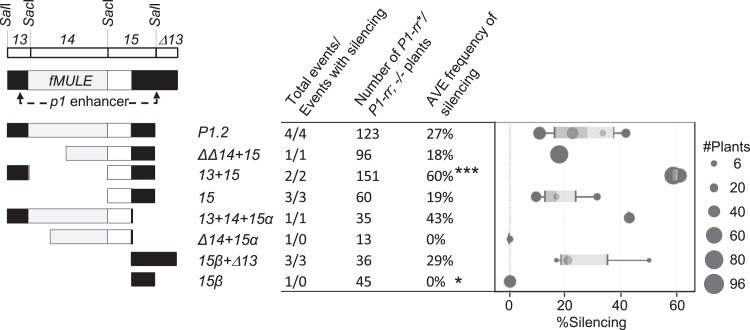
Secondary paramutation of silenced *P1-rr* states induced by transgenes carrying *P1.2* subfragments. A simplified diagram of *P1.2* subfragments is shown on the left of the figure. Location of *p1* cob glume enhancer, *fMULE*, and major restriction sites is shown on top. Shading within transgenic *P1.2* subfragments is similar to Fig. 1; black rectangles indicate sequence of the *p1* cob glume enhancer, light gray rectangles indicate diagram *fMULE*, and open rectangles show DNA that is unique to *p1* locus. The table in the middle of the figure presents a construct level summary for the informative nontransgenic P1-rr*/P1-rr, *−/−* plants (Supplementary Fig. 7). Number of events and plants tested and average frequency of silencing (Materials and methods) for each construct are shown. Three stars indicate statistically significant differences at *P* < 0.0001, and 1 star indicate significant difference at *P* < 0.01 relative the *P1.2* control. *P*-values were calculated by 2-sample test for equality of proportions with Yates’ correction for continuity, followed by Bonferroni–Holm adjustment for multiple comparisons (Materials and methods). The box plot on the right shows silencing frequencies for individual events with each of the dots sized proportionally to the number of transgenic plants assayed for that event. Light- and dark-shaded areas of the box plot indicate 2 middle quartiles with the dividing line indicating median frequency of silencing. The whiskers indicate 1.5 times the minimum and maximum of the respective interquartile range. Additional information regarding segregation of herbicide tolerance and frequencies of strong and weak transgene silencing is shown in Supplementary Fig. 7.

Secondary paramutation, i.e. the ability of a silenced P1-rr* to paramutate a naïve *P1-rr* allele in the absence of the original silencing transgene, is a defining characteristic of paramutation. Secondary paramutation tests were conducted for 8 constructs using families derived from the strongly silenced P1-rr* ears (similar to those in Fig. 2d, e, and h–j). To assay secondary paramutation, transgenic plants were crossed with the nontransgenic *P1-rr* tester (Supplementary Fig. 6), and progeny plants homozygous for *P1-rr* and lacking a transgene were identified and scored (Materials and methods). Results are summarized on a construct level in Fig. 5 with data for individual events shown in Supplementary Fig. 7. For the control *P1.2* construct, secondary paramutation was assayed for 4 transgenic events: the average frequency of silencing was 27%. Among constructs carrying subfragments of *P1.2*, multiple independent transgenic events were tested for *13 + 15* (2), *15* (3), and *15β + Δ13* (3). One construct, *13 + 15*, gave an average frequency of silencing greater than that of *P1.2* control (60%), while 2 constructs, *15* and *15β + Δ13*, exhibited frequencies of silencing not significantly different from *P1.2* control (19 and 29%, respectively). For the remaining 4 constructs (*ΔΔ14 + 15*, *13 + 14 + 15α*, *Δ14 + 15α*, and *15β*), we were able to test only 1 event per construct. From these constructs, only the *15β* construct showed result significantly different from *P1.2* control, with 0% silencing. The *Δ14 + 15α* construct also showed 0% silencing, but in this case, only 13 plants were tested, rendering this result not statistically significant. Finally, the *ΔΔ14 + 15* and the *13 + 14 + 15α* gave moderate frequencies of silencing (18 and 43%, respectively) that were not significantly different from the *P1.2* control. Based on these results, we conclude that sequences required and sufficient for paramutation are located within *13* and *15* subfragments. In accordance with established paramutation terminology, the P1-rr* epialleles silenced by the constructs that induced heritable and secondary paramutagenic silencing will be further designated as *P1-rr′*.

### Distinct “blushed” ear phenotype preferentially induced by constructs lacking subfragment *13*

Initial experiments showed that the *P1.2* transgenes elicited a variety of *P1-rr′* ear phenotypes, ranging from light red to very pale kernel pericarp and cob glumes (Fig. 2b–e). Kernels on silenced ears often exhibited prominent red stripes emanating from the silk scar region (Fig. 2d and e), a phenotype termed “dark crown” by Emerson (Emerson 1917). Analyses of additional transgenic events containing *P1.2* (Sidorenko and Chandler 2008) also identified fully silenced *P1-rr′* states with colorless pericarp and cob glumes (Fig. 2j). Interestingly, in this study using transgenes carrying subfragments of *P1.2*, we observed a distinct pigmentation pattern of “blushed” pericarp, weak cob glume color, and absence of dark crown kernels (Fig. 2f–i). Closer examination of hand sections from representative kernels of patterned and blushed phenotypes revealed differences in pigment deposition within the pericarp. In dark crown patterned kernels, sections through the red stripe revealed pigment accumulation in the crown region and an outer cell layer of pericarp from the kernel gown (side) (Fig. 2k). In contrast, sections of blushed kernels exhibited colorless crown and pigmented inner pericarp cell layers of the kernel gown (Fig. 2l). These distinct pericarp pigmentation patterns coincide with spatial location of epidermal LI and subepidermal LII pericarp cell lineages (Dellaporta et al. 1991) and suggests a possibility that the dark crown pattern is associated with a greater silencing of the inner LII pericarp cell layers, while the blushed phenotype is associated with a greater silencing of the outer LI cell layers.

Analyses of frequencies of blushed ears revealed that in the silencing induction test, the highest occurrence was among the *ΔΔ14 + 15*, *15*, *Δ14 + 15α*, and *15β* constructs (19–45%), while only occasional blushed ears were observed among *13 + 15*, *15α*, and *Pb* constructs (1–5%) (Supplementary Fig. 8); no blushed ears were observed among the *P1.2*, *13 + 14*, *13 + 14 + 15α*, and *15β + Δ13* constructs. In contrast to the relatively high frequency of blushed ears in the induction tests, heritability of the blushed ear phenotypes after transgene segregation was relatively low. Among the 4 constructs with significant blushed ear induction, construct *15* elicited the highest heritability (22%) of blushed ears, while the remaining constructs (*ΔΔ14 + 15*, *Δ14 + 15α*, and *15β*) exhibited low (0–3%) blushed ear heritability. Similarly, frequency of blushed ears was low in secondary paramutation tests (0–17%). In both heritability and secondary paramutation tests, blushed ears (4–17%) were observed in progeny from patterned ears initially silenced by the *P1.2* and *13 + 14 + 15α* constructs, suggesting that variation in silencing phenotype occurred following transgene segregation.

In summary, the distinct blushed ear phenotypes were preferentially induced by constructs lacking subfragment *13*. Moreover, the blushed ear phenotypes were unstable and frequently reverted to red pericarp and cob pigmentation. These observations are consistent with the idea that the blushed ear phenotype results from silencing being predominantly localized to the pericarp LI cell layers. Because the female megasporocyte develops from the LII cells (Dellaporta et al. 1991), a lack of silencing in the LII may account for the low heritability of silencing and frequent reversion of the blushed ear phenotype.

### Silenced *P1-rr′* alleles exhibit transgene-specific DNA methylation patterns

**Fig. 6.**
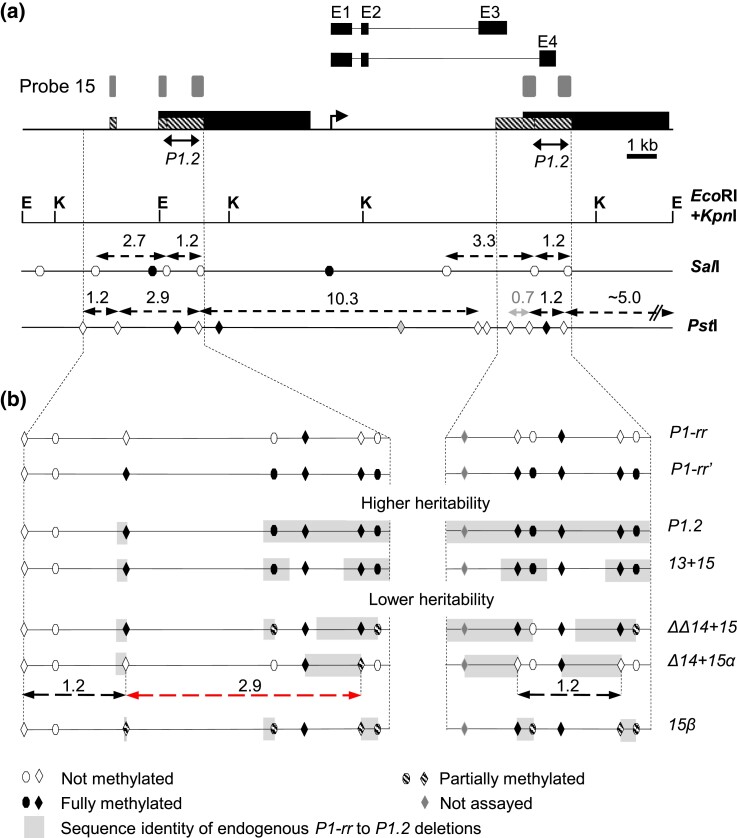
Summary of *Sal*I and *Pst*I methylation within transgene-silenced endogenous *P1-rr′* allele. DNA blots used to generate this summary are shown in Supplementary Figs. 9–11. For both a and b, ovals denote *Sal*I, and diamonds denote *Pst*I restriction sites. *Eco*RI and *Kpn*I are not sensitive to cytosine methylation, while *Sal*I is sensitive to CpG methylation, and *Pst*I is sensitive to CpNpG methylation. Shading of ovals and diamonds indicates methylation status of the *Sal*I and *Pst*I sites: black, complete methylation; striped, partial methylation; and open, no methylation. The 0.7-kb *Pst*I fragment is shown in gray because this fragment was faint on DNA blots and difficult to reliably observe (Supplementary Figs. 9c, 10c, and 11c). a) Map of the endogenous *P1-rr* gene locus. *P1-rr* exons (black boxes) and introns (thin lines) (Grotewold et al. 1991) and location of probe 15 (gray boxes) (Lechelt et al. 1989) are shown above the *P1-rr* map. The *P1-rr* transcription start site is shown as a bent arrow. The 5.2-kb direct repeats (black rectangles) overlap with the smaller 1.2-kb direct repeats (hatched boxes). Below the *P1-rr* map, double-headed black arrows indicate the location of the *P1.2* transgene fragments. The restriction maps of *Eco*RI (E) + *Kpn*I (K), *Sal*I, and *Pst*I sites are also shown below the *P1-rr* map. Double-headed arrows above the *Sal*I and *Pst*I restriction maps indicate fragments recognized by *P1-rr* probe 15. b) The expanded views of *Sal*I and *Pst*I DNA methylation within the upstream and downstream 1.2-kb direct repeats, as detected by hybridization with probe 15. Methylation of the active endogenous *P1-rr* allele and transgene-silenced *P1-rr′* epiallele is shown on top. The names of the transgenic constructs are shown on the right. Double-headed arrows below the *Δ14 + 15α* diagram show locations of the missing 2.9-kb band (red) and observed 1.2-kb doublet (black) (Supplementary Fig. 10c). Gray boxes indicate sequence identity of each transgene to the endogenous *P1-rr* allele.

Prior studies showed that *P1.2*-induced *P1-rr′* paramutation is associated with increased cytosine methylation within the 1.2-kb *P1-rr* enhancer sequences (Sidorenko and Peterson 2001). Specifically, DNA gel blot analyses detected methylation of the *Sal*I restriction sites flanking the endogenous *P1.2* fragment and the corresponding *Sal*I sites within the *P1.2* transgene (Sidorenko and Peterson 2001). DNA blots were also used to assay cytosine methylation of spontaneous *P1-rr* epialleles *P1-pr* and *P1-pr^TP^* (Das and Messing 1994; Sekhon et al. 2012). Here, we used the same approach to assess cytosine methylation of different *P1-rr′* states induced by various transgenes, with a focus on comparing methylation of highly heritable *P1-rr′* (induced by *P1.2* and *13 + 15*) and poorly heritable *P1-rr′* (induced by *ΔΔ14 + 15*, *Δ14 + 15α*, and *15β* transgenes) (Fig. 6; Supplementary Figs. 9–11).

Because biolistic transformation produces transgenic events with complex multicopy structures, we assessed the complexity of transgenic events using restriction enzymes *Eco*RI and *Kpn*I that are insensitive to cytosine methylation. Genomic DNA gel blots were hybridized with *P1-rr* probe 15 which detects both the endogenous *P1-rr* and the transgene(s) (Fig. 6a). The control samples containing active *P1-rr* or heritably silenced *P1-rr′* produced a simple pattern of ∼8.5, 4.0, and 2.5-kb bands (Fig. 6a; Supplementary Figs. 9a, 10a, and 11a). In contrast, transgenic samples produced, in addition to the endogenous *P1-rr-*derived bands, multiple bands of varying intensity indicative of complex multicopy transgene DNA insertions (Supplementary Figs. 9a, 10a, and 11a).

Consistent with prior results, active *P1-rr* and heritably silenced *P1-rr′* epialleles exhibit distinctly different DNA methylation patterns as assessed by *Sal*I and *Pst*I restriction enzyme digestion and hybridization with probe 15 (Fig. 6a; Supplementary Fig. 9b and c). For the active *P1-rr* allele (first lanes), *Sal*I produces bands of 3.3, 2.7, and 1.2 kb (doublet), while *Pst*I produces bands of 10.3, ∼5.0, 2.9, 1.2-kb doublet, and 0.7 kb. The heritably silenced nontransgenic *P1-rr′*, initially induced by *P1.2* and maintained in the absence of an inducing transgene, lacks these bands (second lanes) and exhibits only high molecular weight (>10 kb) bands consistent with methylation of *Sal*I and *Pst*I restriction sites (Fig. 6b). These results are consistent with several prior studies demonstrating hypermethylation of silenced *P1-rr* epialleles, including endogenous *P1-pr* and *P1-pr^TP^* and transgene-induced *P1-rr′* (Das and Messing 1994; Sidorenko and Peterson 2001; Sekhon et al. 2012).

We then assayed methylation levels in 3 strongly silenced and highly heritable *P1-rr′* cases (1 event induced by *P1.2* and 2 events induced by *13 + 15*; Supplementary Fig. 9). *Sal*I digestion produced only high-molecular-weight bands, indicating full methylation of *SalI* restriction sites in both *P1-rr′* and the *P1.2* and *13 + 15* transgene sequences (Supplementary Fig. 9b). *Pst*I digestion produced mostly high-molecular-weight bands, and the ∼5.0-, 2.9-, and 1.2-kb bands were absent (Supplementary Fig. 9c). These results indicate that the *P1.2* and *13 + 15* transgenes induce high levels of methylation of the *Sal*I and *Pst*I restriction sites located within the regions of homology to the transgene (light gray rectangles in Fig. 6b).

Next, we assessed methylation in *P1-rr′* cases with strong silencing but weak heritability, induced by the *ΔΔ14 + 15*, *Δ14 + 15α*, and *15β* constructs (Supplementary Figs. 10 and 11). Maintaining strong *P1-rr* silencing induced by these constructs required using transgenic plants, in which transgenic DNA hybridizing to probe 15 resulted in a more complex banding pattern. Despite this complication, analyses were informative and revealed distinct methylation patterns in these *P1-rr′* events (Fig. 6b).

For the *ΔΔ14 + 15* transgene, 9 plants from 1 event were analyzed. Eight plants had strong silencing phenotype and exhibited faint 3.3-, 2.7-, and 1.2-kb *Sal*I bands (Supplementary Fig. 8b), while there were no detectable ∼5.0-, 2.9-, and 1.2-kb *Pst*I bands (Supplementary Fig. 1c), suggesting partial *Sal*I and full *Pst*I methylation, respectively (Fig. 6b). The remaining plant with weak *P1-rr′* silencing exhibited less methylation as indicated by prominent *Sal*I and *Pst*I bands (black stars in Supplementary Fig. 10b and c).

For the *Δ14 + 15a* transgene, there is no detectable *Sal*I methylation as clear 3.3-, 2.7-, and 1.2-kb bands are present (Supplementary Fig. 10b). This is not surprising because the *Sal*I sites are located outside the region of transgene homology (Fig. 6b). Methylation of *Pst*I sites (Supplementary Fig. 1c), which are located at the edges of the *Δ14 + 15a* construct (Fig. 6b), is more complex: ∼5.0- and 1.2-kb bands are clearly visible, indicating that sites flanking the 1.2-kb *Pst*I fragments are not methylated (black double-headed arrows below *Δ14 + 15a* map in Fig. 6b). The 2.9-kb band is very faint (red arrow in Supplementary Fig. 1c), suggesting that the right-hand *Pst*I site flanking 2.9-kb fragment is predominantly methylated (red double-headed arrow below *Δ14 + 15a* map in Fig. 6b).

For the *15β* transgene, both *Pst*I and *Sal*I sites are located at the ends of the transgene sequence homology and exhibit variable methylation (Fig. 6b). In the *Sal*I digestions, 1 event (3 plants; Supplementary Fig. 11b, left) has 3.3-, 2.7-, and 1.2-kb bands indicating little or no *Sal*I site methylation. The other event (Supplementary Fig. 11b, right) has 3 plants with 3.3-, 2.7-, and 1.2-kb bands (little or no *Sal*I site methylation) and 2 plants in which the 3.3- and 2.7-kb bands are faint, while the 1.2-kb band is missing (red arrows in Supplementary Fig. 11b). In the *Pst*I digestion, 1 event (Supplementary Fig. 11c, left) lacked 2.9- and 1.2-kb bands indicating full *Pst*I site methylation, while the other event (Supplementary Fig. 11c, right) has the ∼5.0-, 2.9-, and 1.2-kb bands in 3 out of 5 tested plants.

To summarize, these results revealed patterns of *P1-rr′* methylation associated with both strength of silencing and degree of heritability. Strong and highly heritable *P1-rr* silencing induced by the *P1.2* and *13 + 15* transgenes correlated with complete methylation of *SalI* and *PstI* sites located within the transgene-homologous sequences. However, strongly silenced but weakly heritable *P1-rr* silencing induced by *ΔΔ14 + 15*, *Δ14 + 15α*, and *15β* transgenes correlated with partial methylation of *Sal*I and *Pst*I sites. In these cases, partially methylated or not methylated restriction sites were located at the ends of the homology to transgene fragments. These results are reminiscent of previous studies showing that methylation of integrated transgenes is restricted to the transgene and does not spread to adjacent sequences (Day et al. 2000).

### Sequences mediating *p1* paramutation are transcribed in both active *P1-rr* and silenced *P1-rr′*

**Fig. 7.**
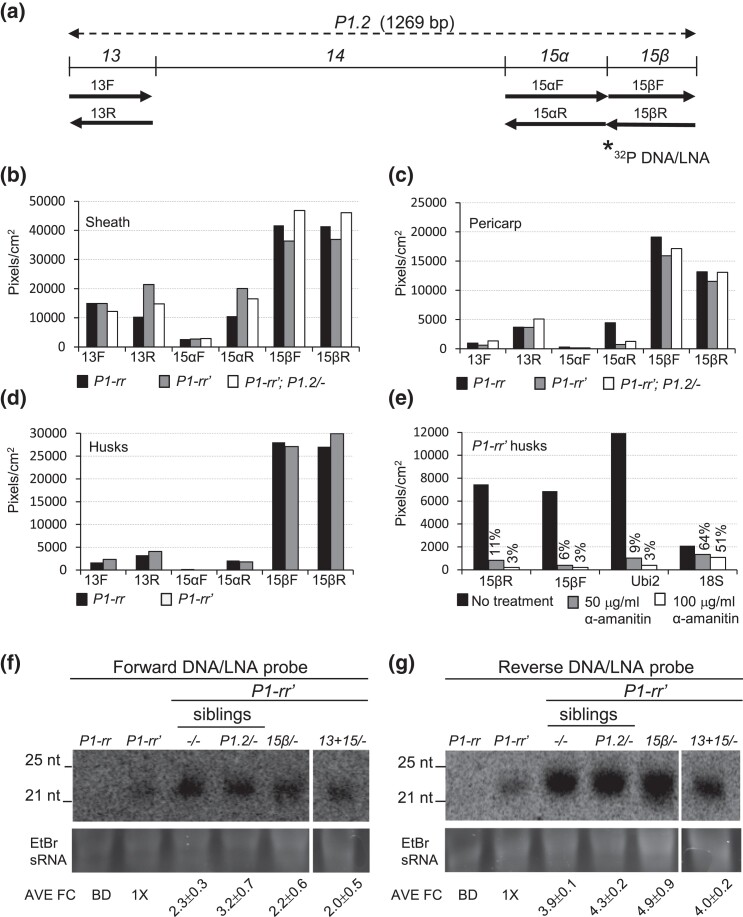
Transcription and sRNA accumulation within the sequences required for *p1* paramutation. a) Diagram of the *P1.2* fragment and the location of probes used in nuclear run-on and sRNA blot analysis. RNA probes used for nuclear run-on analysis are shown as arrows below the *P1.2* map. Complementary 45-nt DNA/LNA oligos used for the northern blot analysis of sRNA is shown by a star. The exact location of the 45-bp DNA/LNA probes is diagrammed in Supplementary Fig. 1a. For b through e, the names of RNA probes used for detection of nuclear run-on transcripts are indicated along the *x*-axis, and the mean signal intensity (pixels/mm^2^) is indicated on the *y*-axis. b) Transcription detected with *13*, *15α*, and *15β* probes in nuclei extracted from young sheath. c) Transcription detected *13*, *15α*, and *15β* probes in nuclei extracted from immature pericarps. d) Transcription detected with *13*, *15α*, and *15β* probes in nuclei extracted from young *P1-rr* and *P1-rr′* husks. e) Effect of α-amanitin on transcription detected with the *15β* probe in nuclei extracted from young *P1-rr′* husks. The percentages of the signal remaining after treatment with 50 µg/ml and 100 µg/ml α-amanitin relative to untreated nuclei are shown above the corresponding bars. f, g) Northern blot analyses of sRNA accumulation in the young ears of active *P1-rr*, silenced *P1-rr′* (no transgene), and transgenic *P1-rr′* plants. Forward and reverse DNA/LNA oligos were end-labeled with ^32^P and used as hybridization probes (Materials and methods). A photo of the gels stained with ethidium bromide illustrates sRNA loading. Location of 25 and 21 nt of the microRNA marker is shown on the right of each blot. The genotype of each sample is indicated on the top of each lane. The sRNA sample in the first lane was extracted from young *P1-rr* ears. The sRNA sample in lane 2 was from a silenced patterned *P1-rr′* that was propagated in the absence of *P1.2* transgene for 10 consecutive generations. The sRNA samples loaded in lanes 3 and 4 were from sibling *P1-rr′* plants that were homozygous for *P1-rr*, but differed in *P1.2* transgene presence/absence: nontransgenic sibling (lane 3) and transgenic sibling (lane 4). The last 2 lanes contain samples from strongly silenced homozygous *P1-rr′* plants that were hemizygous for the *15β* and *13 + 15* transgenes. Because sRNA levels in the *P1-rr* sample were below detection (indicated as BD), the average pixel volumes of *P1-rr′* (lane 2) were used to calculate fold change (FC) in lanes 3–6. Average FC relative to *P1-rr′* was calculated from 2 technical replicates and is shown below each lane.

Transcription by DNA-dependent RNA polymerases has been shown to play key roles in the initiation, establishment, and maintenance of sRNA-mediated transcriptional silencing (Haag and Pikaard 2011; Nuthikattu et al. 2013; Sasaki et al. 2014). Transcription of the *p1* paramutagenic sequences may generate RNA transcripts converted to dsRNA through the RDR activity of MOP1 (Sidorenko and Chandler 2008) and subsequently processed to sRNAs. To test whether paramutagenic sequences are transcribed, we performed run-on transcription assays using nuclei prepared from young sheaths and developing pericarps of active *P1-rr* of the silenced nontransgenic *P1-rr′* and transgenic *P1-rr′; P1.2/−* genotypes (Fig. 7b and c) and young husks of active *P1-rr* and silenced nontransgenic *P1-rr′* (Fig. 7d). Assays were performed in the presence of ^32^P-labeled ribonucleotides, and the resulting ^32^P-labeled transcripts were hybridized to RNA probes immobilized on nylon membrane corresponding to the *13*, *15α*, and *15β* subfragments (Fig. 6a; Materials and methods).

The results show that the sequences required for *p1* paramutation produced transcripts that hybridize with both forward and reverse probes in all assayed tissues (Fig. 6b–d). The highest transcription levels were observed for the *15β* forward and reverse probes, indicating that both DNA strands were strongly transcribed. Strikingly, all tested genotypes had roughly similar transcription levels in all tissues, suggesting that transcription was not reduced in the silenced nontransgenic *P1-rr′* and the transgenic *P1-rr′; P1.2/−* genotypes. This finding contrasts with transcription of the *p1* coding sequence which is reduced ∼5X in the silenced *P1-rr′* allele relative to the active *P1-rr* (Sidorenko and Chandler 2008). The results showing similar levels of transcription in the active *P1-rr* and silenced *P1-rr′* regulatory sequences are reminiscent of the results at the maize *b1* locus where the sequences required for paramutation were transcribed in the active and transcriptionally silenced *b1* alleles at similar levels (Alleman et al. 2006).

### α-Amanitin sensitivity implicates RNA Pol-II in transcription of the *p1* paramutation sequences

To determine which of the 5 plant DNA-dependent RNA polymerases is responsible for transcription of the *p1* paramutagenic sequences, we tested the effects of the fungal toxin α-amanitin, which most strongly inhibits the Pol II polymerase, even at low doses of 5–10 µg/ml (Strain et al. 1971; Haaf and Ward 1996; Haag et al. 2012). Higher doses of α-amanitin (50–100 µg/ml) suppress Pol III activity (Haeusler and Engelke 2006), while Pol I remains largely insensitive to α-amanitin even at high concentrations (De Lange et al. 1985). The remaining 2 plant-specific polymerases involved in RdDM, Pol IV and Pol V, were demonstrated to be insensitive to α-amanitin in vitro (Haag et al. 2012). We assayed the effect of α-amanitin on transcription from the *15β* subfragment in young husks of heritably silenced *P1-rr′* plants. Our results revealed a ∼10- and ∼33-fold reduction of transcription within the *15β* subfragment in the nuclei treated with 50 and 100 µg/ml of α-amanitin, respectively (Fig. 7e). These levels of transcriptional suppression were similar to those of the Pol II-transcribed maize *Ubiquitin2* control (Fig. 7e). As expected, transcription of Pol I-transcribed *18S* control was less sensitive to α-amanitin; ∼2-fold reduction was observed in the presence of these levels of α-amanitin. Based on the high α-amanitin sensitivity of the *15β* transcripts, we conclude that Pol-II RNA polymerase is most likely responsible for transcription of the *p1* paramutagenic sequences.

### sRNAs are more abundant in *P1-rr′* than *P1-rr*

Genetic tests implicated the involvement of the RNA-mediated DNA methylation pathway in *p1* paramutation (Sidorenko and Chandler 2008; Sidorenko et al. 2009). To further investigate the role of this pathway in *p1* paramutation, we assayed accumulation of sRNAs derived from the *p1* paramutagenic sequences. Initial experiments failed to detect sRNA in either *P1-rr* or *P1-rr′* using RNA blots containing 20 µg of total RNA from pericarp or leaf tissues and hybridized with randomly ^32^P-labeled *13*, *14*, and *15* DNA probes (Sidorenko, data not shown). Next, we used a highly sensitive detection method that was developed for assaying low-abundance sRNAs involved in *b1* paramutation (Arteaga-Vazquez et al. 2010). Total RNA was extracted from sRNA-rich young ears, and low-molecular-weight RNAs were enriched (Materials and methods) and loaded in high amounts (100 µg per lane). The resulting blots were hybridized with ^32^P end-labeled with highly sensitive DNA/LNA oligos (Fig. 4a; the exact location of the DNA/LNA oligos is shown in Supplementary Fig. 1a). sRNA blots hybridized with the complementary forward (F) and reverse (R) DNA/LNA oligos are shown in Fig. 7f and g. and sRNA signal was not detected in active *P1-rr* (1st lanes), while varying levels of sRNA were observed in the silenced *P1-rr′* samples (2nd and 3rd lanes). Specifically, low levels of sRNA were detected in samples from a *P1-rr′* lineage that was maintained in the absence of the inducing transgene for 10 consecutive generations (2nd lanes). Relatively this *P1-rr′* lineage, higher sRNA levels (2.3- to 3.9-fold) were observed for *P1-rr′* that had segregated away from the *P1.2* transgene in the prior generation (3rd lanes). Interestingly, sRNA levels in the sample from transgenic *P1-rr′*; *P1.2/−* (4th lanes) were similar to that of a nontransgenic *P1-rr′* sibling (3rd lanes). Elevated sRNA levels (2.2- to 4.9-fold) were also detected in samples from the silenced *P1-rr′* plants containing the *15β* and *13 + 15* transgenes (last 2 lanes). In summary, using large quantities of enriched sRNA and highly sensitive DNA/LNA probes enabled detection of increased sRNA levels in nontransgenic and transgenic *P1-rr′* samples relative to the active *P1-rr* for which sRNAs were not detected.

## Discussion

### Minimal *p1* paramutagenic sequence is ∼600 bp and overlaps with a *P1-rr* enhancer

A previous study had shown that a 1.2-kb *p1* noncoding fragment (*P1.2*) could induce paramutation of *P1-rr* (Sidorenko and Peterson 2001; Sidorenko and Chandler 2008). Here, we used transgenic maize plants to identify the minimal sequences within the *P1.2* fragment required for *p1* paramutation. The results showed that a 409-bp fragment (*15*) was sufficient to induce frequent and strong *P1-rr* silencing. However, the resulting *P1-rr*-silenced states were less heritable than those induced by the full-length *P1.2* fragment. Heritability and secondary paramutation were more frequent in plants containing *15* plus an additional 202 bp of *13* subfragment (Supplementary Fig. 1b). While it remains unclear how addition of the *13* subfragment improves heritability and secondary paramutagenicity of the *P1-rr′* state, one possibility may be that transgenes carrying both *15* and *13* subfragments produce sRNAs from the entire required for paramutation *p1* enhancer sequence (Fig. 1b), establishing DNA methylation and repressive chromatin that is more effectively inherited and transmitted to the naïve *P1-rr* in the absence of the silencing transgene. In contrast, the inclusion of sequences within subfragment *14* did not increase *P1-rr* silencing, heritability, or secondary paramutation of *P1-rr* silenced states. Thus, our results identify a minimal ∼600-bp sequence within *P1.2* sufficient to induce *p1* paramutation that is heritable and can mediate secondary paramutation at high frequencies.

Prior studies of *P1-rr* gene structure revealed that the upstream *P1.2* fragment has properties of a transcriptional enhancer (Sidorenko et al. 2000; Sidorenko and Peterson 2001). Analysis of related *P1-rw* alleles identified a 386-bp sequence overlapping *P1.2* (Fig. 1a) that acts as an enhancer of *p1* expression in cob glumes (Zhang and Peterson 2005; Zhang et al. 2006). In *P1-rr*, the 386-bp cob glume enhancer sequence overlaps with minimal paramutagenic *P1.2* subfragments *13* and *15* (Fig. 1b; Supplementary Fig. 1a). This overlap suggests that the *p1* enhancer and paramutation determinants are functionally related. The involvement of enhancer sequences in paramutation is well established for the maize *b1* gene, which regulates plant anthocyanin accumulation. An enhancer of *b1* expression is located ∼100-kb upstream of the *b1* coding sequence; this long-distance enhancer consists of seven 853-bp tandem (hepta) repeats (Stam, Belele, Dorweiler, et al. 2002; Stam, Belele, Ramakrishna, et al. 2002) and is required for both high-level expression and paramutation. Moreover, the *b1* hepta repeats mediate high expression of a heterologous reporter gene in transgenic plants and also induce strong paramutation of a susceptible endogenous *b1* allele in multiple transgenic events (Belele et al. 2013). Finally, genetic analyses of the maize *pl1* gene indicated a similar association between paramutation and a 3′ enhancer of *pl1* expression (Erhard et al. 2013; Hollick 2017). Together, the results from *p1*, *b1*, and *pl1* studies strongly suggest that enhancer sequences mediate both high expression and paramutation of these maize genes.

### *P1-rr* paramutagenic sequences are present in multicopy repeats

The paramutable *P1-rr* allele and the paramutagenic epialleles *P1-rr′* and *P1-pr* have identical locus structures (Das and Messing 1993; Sidorenko and Peterson 2001) and contain 4 copies of the ∼600-bp paramutagenic sequence within the 1.2-kb direct repeats flanking the *p1* coding sequence (Fig. 1a), whereas, a nonexpressing *p1-ww* allele that is neutral to paramutation has a single truncated copy of the 1.2-kb repeat (Sidorenko and Peterson 2001; Goettel and Messing 2013b). However, other alleles show that the presence of *P1.2* repeats is not sufficient to induce paramutation: for example, the spontaneous *P1-pr^TP^* isolate is structurally identical to *P1-rr* but is not paramutagenic (Sekhon et al. 2007; Sekhon and Chopra 2009). Furthermore, the *P1-wr* alleles contain multiple repeats of sequences highly similar to *P1.2* (97%, not shown), but they do not participate in paramutation (Sidorenko and Peterson 2001; Goettel and Messing 2009). These observations suggest that *p1* paramutation may require specific epigenetic states of the paramutagenic repeated sequences.

One epigenetic chromatin mark found associated with the *P1.2* repeat sequences in the paramutagenic *P1-rr′* and *P1-pr* epialleles is presence of increased nonsymmetric CHH cytosine methylation (Sekhon et al. 2012; Goettel and Messing 2013b), in addition to high CG and CHG methylation. The *P1.2* repeats of the *P1-pr^TP^* and *P1-wr* alleles that do not participate in paramutation have significant CG and CHG methylation, but they have low CHH methylation (Sekhon and Chopra 2009; Sekhon et al. 2012). Because CHH methylation is established via sRNA-mediated mechanisms (Matzke and Mosher 2014), this finding is consistent with the model that RdDM pathways are involved in marking the *P1.2* repeat sequences as a result of paramutation (Sidorenko and Chandler 2008; Sidorenko et al. 2009).

In addition to *p1*, a link between sequence repeats and paramutation has been extensively documented at other maize loci (Chandler et al. 2000; Chandler and Stam 2004; Stam 2009; Hovel et al. 2015; Hollick 2017). At the *r1* locus, genetic studies demonstrated that alleles with 4 *r1* gene copies were strongly paramutagenic, while alleles with fewer gene copies exhibited reduced paramutagenicity; alleles with a single *r1* gene copy were not paramutagenic (Kermicle et al. 1995; Panavas et al. 1999). At the *b1* locus, analysis of recombinant alleles differing in number of 853-bp enhancer repeats showed that paramutagenicity was directly proportional to repeat copy number (Stam, Belele, Dorweiler, et al. 2002). These results from the maize *r1* and *b1* loci strongly link repeat number with strength of paramutation, while the involvement of repeat sequences has so far not been reported for maize *pl1* paramutation (Hollick 2017).

### *fMULE* sequences are not likely to play a significant role in transgene-induced *p1* paramutation

The *P1-rr* gene locus harbors multiple transposable elements within its flanking 5.2-kb direct repeats (Fig. 1a), the proximal *Pb* promoter sequences and introns (Goettel and Messing 2010). Some of these elements, specifically *fMULE* that are present within the distal *P1.2* enhancer and proximal promoter fragment, were proposed to play regulatory roles in spontaneous silencing and paramutation of the endogenous *P1-rr* allele (Goettel and Messing 2013a). In the proposed model (Goettel and Messing 2013a), sRNA-mediated silencing of *MULEs* at other genomic locations affects *fMULEs* within the *P1-rr* regulatory sequences *in trans* by a homology-based sRNA-mediated silencing mechanism. Silencing of *fMULEs* within the *P1-rr* allele may then lead to spreading of silenced chromatin to the nearby *P1-rr* enhancer, resulting in establishment, and later maintenance, of heritable transcriptional *P1-rr* silencing (Goettel and Messing 2013b). Our experiments, however, did not reveal a significant role for *MULE* sequences in transgene-induced *P1-rr* paramutation. Specifically, subfragment *14*, containing an *fMULE* sequence (Goettel and Messing 2013a), was neither required nor sufficient for strong *P1-rr* silencing and paramutation. Similarly, fragment *Pb* containing a 145-bp *MULE* sequence (not shown) induced only phenotypically weak *P1-rr* silencing. Rather, our experiments indicate a critical role for the enhancer sequences located within the *P1-rr* locus subfragments *15* and *13*. These sequences do not contain significant similarities to TEs nor to the enhancer sequences involved in *b1* paramutation (Sidorenko, not shown).

### Sequences required for *p1* paramutation are transcribed by Pol II RNA polymerase

Nuclear run-on assays revealed that the paramutagenic sequences are transcribed in all tested tissues (young sheath, husks, and pericarp) and in all tested genotypes (active *P1-rr* and silenced *P1-rr′* with and without the *P1.2* transgene). Transcription is strongly inhibited by moderate doses of α-amanitin, indicating that RNA Pol II is most likely responsible. Although transcripts hybridizing to forward and reverse probes were detected, it remains unclear whether Pol II transcription is bidirectional or if Pol II produces transcripts from 1 strand that serve as a template for RDR-mediated transcription to generate complementary RNA. These results at *p1* are similar to those at *b1*, where α-amanitin-sensitive transcription from both strands of the *b1* enhancer was detected in samples from both paramutagenic and paramutable alleles (Alleman et al. 2006; Arteaga-Vazquez et al. 2010).

At present, it is not clear how Pol II transcription may regulate paramutation. One possibility is that Pol II-derived transcripts are converted by RDR to dsRNA, which can be processed to sRNAs that can target cytosine methylation and repressive chromatin modifications to the corresponding sequences. In *Arabidopsis*, expression-dependent silencing of active exogenous transposons exemplifies a functional link between Pol II-derived TE transcripts and RDR6-dependent sRNA production and DNA methylation (Nuthikattu et al. 2013; Fultz et al. 2015; McCue et al. 2015; Panda et al. 2016; Fultz and Slotkin 2017). However, because there is no published evidence that suggests involvement of the maize RDR6 orthologs in paramutation, it remains unclear whether a similar mechanism may act at the *P1-rr* enhancer sequences. Another possibility is that transcription rather than transcripts may play a role in regulation of enhancer activity and paramutation. A precedent for a role of transcription in gene regulation was described for a human erythroid cell line where expression of the endogenous *Cdkn1b* gene depended on transcription per se, not transcripts from a downstream long noncoding RNA (Paralkar et al. 2016). Further investigation will be required to discern the role of transcripts and transcription in the mechanism of *p1* paramutation.

### *P1-rr* paramutation is associated with increased levels of sRNAs and cytosine methylation of the *p1* paramutagenic sequences

Previous genetic results indicated that sRNA-mediated RdDM-like mechanisms are involved in the regulation of *p1* paramutation (Sidorenko and Chandler 2008; Sidorenko et al. 2009). Here we confirmed the presence of sRNAs homologous to the paramutagenic *p1* enhancer sequences and showed that these sRNAs accumulate to higher levels in silenced *P1-rr′* than in *P1-rr.* Interestingly, sRNAs corresponding to the *p1* enhancer repeats are in low abundance, and their detection by Northern blot required loading high amounts of sRNA and probing with sensitive DNA/LNA oligos. The low abundance of sRNAs is similar to that reported for *b1* paramutation, where sRNAs corresponding to the *b1* enhancer repeats were also in low abundance (Arteaga-Vazquez et al. 2010). However, in contrast to *b1* paramutation where similar sRNA levels were detected for paramutagenic and paramutable genotypes (Arteaga-Vazquez et al. 2010), in our experiments, silenced *P1-rr′* exhibited higher sRNA levels than the active *P1-rr,* for which sRNAs were below detection. Notably, sRNA levels were further increased (2.2- to 4.9-fold) in transgenic *P1.2*, *15β*, and *13 + 15 P1-rr′* plants. Similar increases of sRNA levels (∼2- to 3-fold) were also detected for *b1* transgenic plants carrying hepta repeats of paramutagenic sequence (Belele et al. 2013). However, at *b1*, levels of sRNA were reduced in nontransgenic siblings, when we did not detect a reduction of sRNAs in a nontransgenic sample. While additional studies are needed to better understand reasons for the observed differences between *p1* and *b1* paramutation, together, these genetic and molecular results provide additional support for models of sRNA-mediated mechanisms of maize paramutation (Arteaga-Vazquez and Chandler 2010; Hollick 2017).

DNA blot analyses of silenced *P1-rr′* plants revealed increased cytosine methylation of the *Sal*I and *Pst*I restriction sites relative to *P1-rr*, which is consistent with prior findings (Das and Messing 1994; Sidorenko and Peterson 2001; Sekhon et al. 2012; Wang et al. 2017). The new insights obtained in this study revealed that the extent of methylation of endogenous *P1-rr′* largely depended on sequences present within the silencing inducing transgene. Specifically, *P1.2* and *13 + 15* that induce highly heritable *P1-rr′* fully encompass the assayed endogenous *P1-rr Sal*I and *Pst*I restriction sites, and these sites were fully methylated in *P1-rr′*. At the same time, *Δ14 + 15α*, *ΔΔ14 + 15*, and *15β* that induce weakly heritable *P1-rr′* have many of the assayed sites located at the edges of sequence identity, and these sites were variably methylated. Reduced methylation of the restriction sites located at the edges of sequence identity between *P1-rr* and transgenes is not surprising and is similar to the results of prior studies (Sekhon et al. 2012) that showed reduced cytosine methylation of the silenced endogenous *P1-rr′* enhancer near the borders of homology to a full-length transgenic *P1.2* fragment that was used to induce paramutation. Accounting for the demonstrated role of RdDM in *p1* paramutation (Sidorenko and Chandler 2008; Sidorenko et al. 2009), these results are consistent with the hypothesis that sRNAs produced from transgenic *P1.2* subfragments drive increased DNA methylation of homologous *P1-rr* enhancer sequences resulting in enhancer inactivation and transcriptional *P1-rr* silencing (Sidorenko and Chandler 2008). The involvement of DNA methylation in transgene-induced *P1-rr* paramutation is similar to its proposed role in paramutation at the *b1* and *r1* loci. Specifically, at the *b1* locus, both endogenous and transgene-induced paramutation correlates with increased cytosine methylation within the tandem hepta repeats required for paramutation (Haring et al. 2010; Belele et al. 2013). At the *r1* locus, paramutation is associated with increased cytosine methylation within specific regulatory sequences of a paramutable *r1* gene (Walker 1998; Panavas et al. 1999; Walker and Panavas 2001). Together, results from *r1*, *b1*, and *p1* support involvement of DNA methylation of enhancer sequences in regulation of endogenous and transgene-induced paramutation in maize, although a causal relationship has not been established.

### Summary and outlook

This study in transgenic plants identifies a minimal ∼600-bp sequence required to induce *p1* paramutation. This sequence occurs in multiple direct repeats near the *p1* gene, overlaps with a previously identified *P1-rr* cob glume enhancer, and is likely transcribed by Pol II RNA polymerase. Transgene-induced paramutation of *P1-rr* is correlated with increased levels of sRNAs and cytosine methylation of the endogenous *P1-rr′* corresponding to the paramutation-inducing sequence. We conclude that this sequence plays important roles in both paramutation and *P1-rr* transcriptional regulation. The identification of the minimal *p1* paramutation sequence will facilitate further biochemical and genetic experiments to identify *trans*-acting factors mediating *P1-rr* enhancer function and paramutation.

## Supplementary Material

iyad178_Supplementary_Data

## Data Availability

Transgenic maize seeds will be available upon request. The authors affirm that all data necessary for confirming the conclusions of the article are present within the article, figures, and supplemental figures. Supplemental material available at GENETICS online.
