# Supplementary material for: Transcribed enhancer sequences are required for maize p1 paramutation
Source: Genetics. 2024 Jan 3;226(1):iyad178. doi: 10.1093/genetics/iyad178 (PMC10763531; doi:10.1093/genetics/iyad178)
Supplement: iyad178_Supplementary_Data [file iyad178_supplementary_data.zip › Figures_S2-S11_GENETICS-2023-306435.pdf]

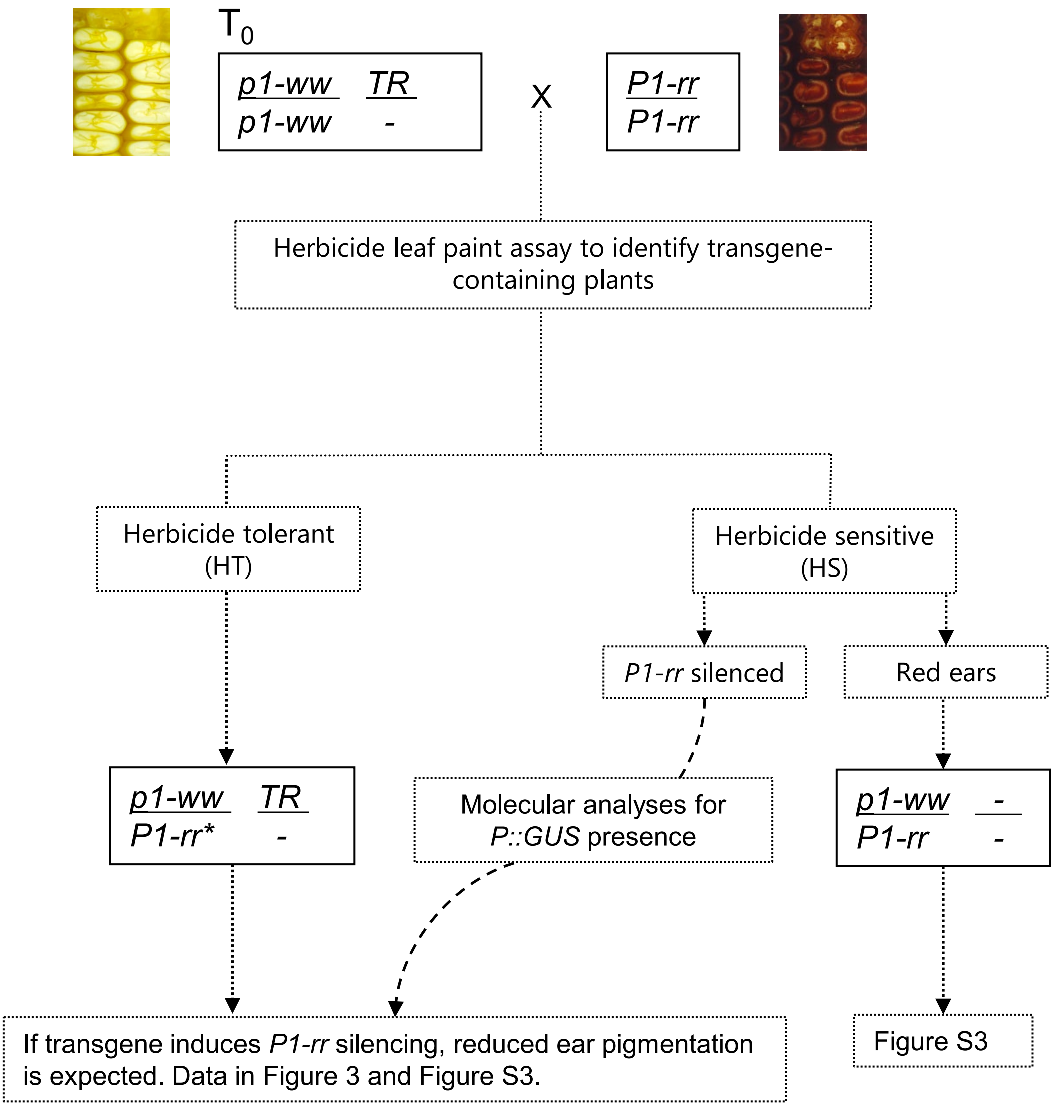


**Figure S2. Crossing scheme for testing transgene-induced silencing of the endogenous *P1-rr* gene.** To assay silencing effects of the transgenes, primary transgenic plants ($T_0$) were crossed with naïve *P1-rr* (Materials and Methods). Representative ear phenotypes of $T_0$ transgenic ears and naïve *P1-rr* are shown on top. The resulting progeny plants were screened by herbicide leaf paint assay (Materials and Methods). Some herbicide sensitive (HS) plants exhibited silenced *P1-rr* ears. Molecular screening of these plants confirmed presence of a *P::GUS* transgene (Figure S3) suggesting silencing of the selectable marker (Materials and Methods). An asterisk (*) indicates exposure of *P1-rr* to a transgene.

| Construct | Event | # of plants HT | # of plants HS | Deviation from 1HT:1HS segreg. | # of transgenic HS plants | # of transgenic *p1-ww/P-rr*; TR/-* plants | Molecular analyses: Probe | # of bands | Est. copy # |
|---|---|---|---|---|---|---|---|---|---|
| *P1.2* | P2P147-28* | 24 | 10 | | 3 | 27 | P15 | 12/0 | 30/0 |
| | P2P147-6 | 23 | 13 | | 3 | 26 | P15 | 6 | 23 |
| | P2P147-4 | 13 | 10 | | 3 | 16 | P15 | 7 | 16 |
| | P2P147-37 | 36 | 10 | HT↑ | 4 | 40 | P15 | 10 | 23 |
| | P2P147-23* | 19 | 1 | HT↑ | | 19 | P15 | 8/0 | 29/0 |
| | P2P147-30 | 8 | 6 | | | 8 | GUS | 6 | na |
| | P2P147-13 | 18 | 2 | HT↑ | | 18 | GUS | 11 | na |
| | P2P147-8 * | 40 | 10 | HT↑ | 3 | 43 | P15 | 11/0 | 163/0 |
| | P2P147-18* | 18 | 2 | HT↑ | | 18 | P15 | 14/0 | 36/0 |
| *13+14* | P2P36-199 | 11 | 58 | HT↓ | 2 | 13 | GUS | 11 | na |
| | P2p36-265 | 4 | 45 | HT↓ | | 4 | GUS | 11 | na |
| | P2P36-222 | 19 | 105 | HT↓ | | 19 | GUS | 13 | na |
| | P2P36-2 | 24 | 27 | | | 24 | GUS | 5 | na |
| | P2P36-191 | 35 | 46 | | 9 | 44 | GUS | 12 | na |
| *ΔΔ 14+15* | P2P104-20 | 3 | 0 | | | 3 | P15 | 8 | 27 |
| | P2P104-21 | 43 | 59 | | 3 | 46 | P15 | 5 | 3 |
| | P2P104-44* | 24 | 3 | HT↑ | 2 | 26 | P15 | 8/0 | 15/0 |
| *13+15* | P2P257-55 | 2 | 8 | | | 2 | P15 | 13 | 11 |
| | P2P257-36 | 17 | 18 | | | 17 | P15 | 8 | 9 |
| | P2P257-21 | 2 | 23 | HT↓ | | 2 | P15 | 10 | 9 |
| | P2P257-42 | 17 | 16 | | | 17 | P15 | 12 | 29 |
| | P2P257-53 | 30 | 22 | | | 30 | P15 | 20 | 75 |
| | P2P257-33 | 17 | 11 | | | 17 | P15 | 12 | 11 |
| | P2P257-37 | 11 | 28 | | | 11 | P15 | 9 | 18 |
| | P2P257-22 | 23 | 11 | | | 23 | P15 | 8 | 5 |
| *15* | P2P62-84 | 12 | 22 | | 1 | 13 | GUS | 15 | na |
| | P2P62-37 | 20 | 17 | | 2 | 22 | GUS | 6 | na |
| | P2P62-47 | 6 | 9 | | | 6 | GUS | na | na |
| | P2P62-52 | 20 | 21 | | | 20 | GUS | 12 | na |
| | P2P62-2 | 67 | 39 | | 2 | 69 | P15 | 8 | 37 |
| | P2P62-11 | 45 | 13 | HT↑ | 3 | 48 | GUS | 9 | na |
| | P2P62-81 | 9 | 16 | | | 9 | GUS | 11 | na |
| *13+14+15α* | P2P146-12* | 42 | 12 | HT↑ | | 42 | P15 | 6/4 | 15/2 |
| | P2P146-5 | 23 | 16 | | 4 | 27 | GUS | 8 | na |
| | P2p146-8 | 6 | 7 | | | 6 | P15 | 12 | 41 |
| | P2P146-10 | 7 | 10 | | | 7 | P15 | 12 | 14 |
| | P2P146-9 | 22 | 14 | | | 22 | P15 | 8 | 11 |
| *Δ 14+15α* | P2P105-12 | 7 | 6 | | | 7 | P15 | 13 | 23 |
| | P2p105-1 | 7 | 2 | | | 7 | P15 | 14 | 20 |
| | P2P105-18 | 3 | 6 | | | 3 | P15 | 8 | 8 |
| | P2P105-10 | 9 | 1 | | 1 | 10 | P15 | 14 | 10 |
| | P2P105-15 | 12 | 10 | | 2 | 14 | P15 | 17 | 66 |
| | P2P105-19 | 14 | 5 | | 1 | 15 | P15 | 11 | 13 |
| | P2P105-14 | 17 | 5 | | 1 | 18 | P15 | 7 | 15 |
| | P2P105-32 | 27 | 1 | HT↑ | | 27 | P15 | 5 | 7 |
| | P2P105-11 | 8 | 4 | | | 8 | P15 | 6 | 9 |
| | P2P105-8 | 10 | 3 | | | 10 | P15 | 8 | 17 |
| | P2P105-7 | 12 | 5 | | | 12 | P15 | 8 | 10 |
| | P2P105-6 | 9 | 3 | | | 9 | P15 | 6 | 2 |
| *15β+Δ 13* | P2P151-20 | 14 | 18 | | 14 | 28 | P15 | 14 | 36 |
| | P2P151-32 | 16 | 14 | | | 16 | P15 | 7 | 6 |
| | P2P151-8 | 24 | 16 | | | 24 | P15 | 8 | 23 |
| | P2P151-4 | 12 | 24 | | 1 | 13 | P15 | 13 | 16 |
| | P2P151-30 | 10 | 11 | | | 10 | P15 | 5 | 4 |
| *15α* | P2P165-6 | 8 | 8 | | | 8 | GUS | 13 | na |
| | P2P165-72 | 12 | 1 | HT↑ | | 12 | GUS | na | na |
| | P2P165-22 | 12 | 5 | | | 12 | GUS | 15 | na |
| | P2P165-27 | 7 | 12 | | | 7 | GUS | 8 | na |
| | P2P165-9 | 22 | 63 | HT↓ | 8 | 30 | GUS | 8 | na |
| | P2P165-38 | 9 | 14 | | | 9 | GUS | 8 | na |
| | P2P165-20 | 12 | 18 | | | 12 | GUS | 13 | na |
| | P2P165-10 | 15 | 36 | HT↓ | | 15 | GUS | 12 | na |
| *15β* | P2P152-12 | 34 | 31 | | 17 | 51 | P15 | 10 | 14 |
| | P2P152-21 | 26 | 18 | | 2 | 28 | P15 | 6 | 9 |
| | P2P152-9 | 34 | 22 | | | 34 | P15 | 11 | 15 |
| | P2P152-22 | 22 | 5 | HT↑ | | 22 | P15 | 9 | 16 |
| | P2P152-1 | 18 | 15 | | | 18 | P15 | 11 | 24 |
| | P2P152-4 | 39 | 33 | | | 39 | P15 | 6 | 12 |
| | P2P152-36* | 19 | 0 | HT↑ | | 19 | P15 | 6/6 | 17/7 |
| | P2P152-2 | 25 | 18 | | | 25 | P15 | 7 | 3 |
| | P2P152-23 | 20 | 16 | | | 20 | P15 | 5 | 4 |
| *Pb* | P2P108-2 | 19 | 106 | HT↓ | 2 | 21 | GUS | 11 | na |
| | P2P108-6 | 24 | 56 | HT↓ | | 24 | GUS | 10 | na |
| | P2P108-3 | 28 | 103 | HT↓ | | 28 | GUS | 10 | na |
| | P2P108-9 | 71 | 21 | HT↑ | | 71 | GUS | 6 | na |
| | P2P108-11 | 28 | 104 | HT↓ | | 28 | GUS | 7 | na |
| | P2P108-1 | 17 | 50 | HT↓ | | 17 | GUS | 6 | na |
| | | | | | 93 | 1585 | | | |

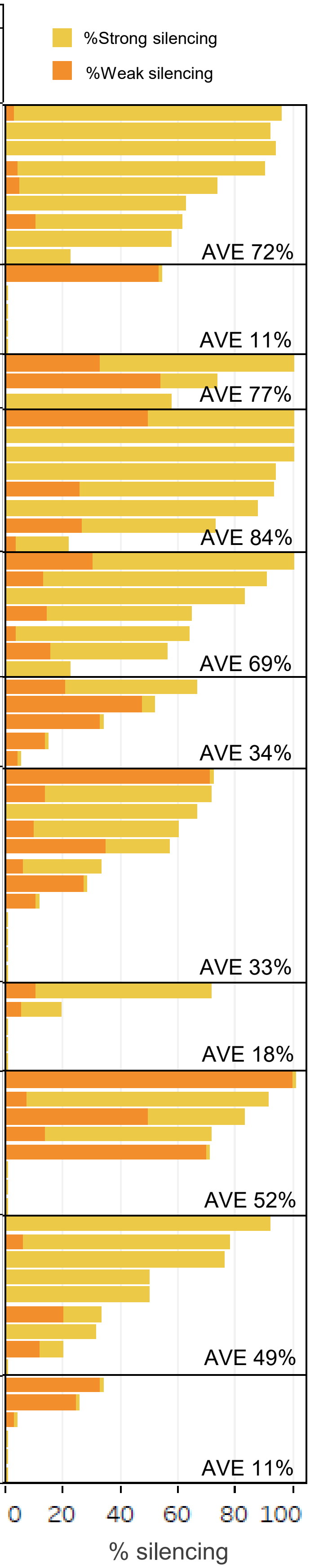


**Figure S3**

**Figure S3. Results of genetic test for induction of *P1-rr* silencing by transgenes carrying *P1.2* sub-fragments**. Construct and event names are shown in the first two columns. Red stars indicate events with more than one segregating transgene insertion. The next two columns show numbers of herbicide tolerant (HT) and herbicide sensitive (HS) plants as determined by the herbicide leaf paint assay (Materials and Methods). The fifth column shows results of $\chi^2$ test. HS and HT plants were expected to segregate at 1:1 ratio. To test this expectation, $\chi^2$ test with the null hypothesis that both classes occurred at equal frequency was conducted. The null hypothesis was rejected when $\chi^2$ with one degree of freedom was greater than 3.84 (P=0.05). Events with greater than expected number of HT plants are indicated as HT↑ while events with lower-than-expected number of HT plants are indicated as HT↓. The sixth column shows the number of HS plants that carried a *P::GUS* transgene, suggesting selectable marker silencing (Materials and Methods). Seventh column shows the number of *p1-ww/P1-rr*; TR/-* transgenic plants calculated as a sum of HT plants and HS plants that were confirmed to carry *P::GUS* transgene in molecular tests. The last three data columns show results of DNA blot analyses of transgenic events; probe name, number of discrete bands, and estimated copy number with probe 15 (abbreviated to P15). For GUS probe, which recognized only transgenic DNA, number of transgenic bands is shown. For probe 15, which hybridizes to transgenes containing *15, 15*$\alpha$, or *15*$\beta$ and the endogenous *p1* gene, count of bands includes both transgenic and endogenous bands. When probe 15 was used, *P::GUS* transgene copy number was estimated by densitometry relative to the endogenous *p1* bands (Materials and Methods). For events with more than one transgenic locus, *P::GUS* number of bands and estimated copy numbers in segregating plants with different banding patterns are shown with a slash. The bar chart on the right shows frequencies of weak (orange) and strong (yellow) *P1-rr* silencing for each transgenic event. To improve comprehension of differences between constructs, transgenic events within each construct were sorted from high to low frequency of *P1-rr* silencing. Average frequency of silencing (also in Figure 3) is shown for convenience.

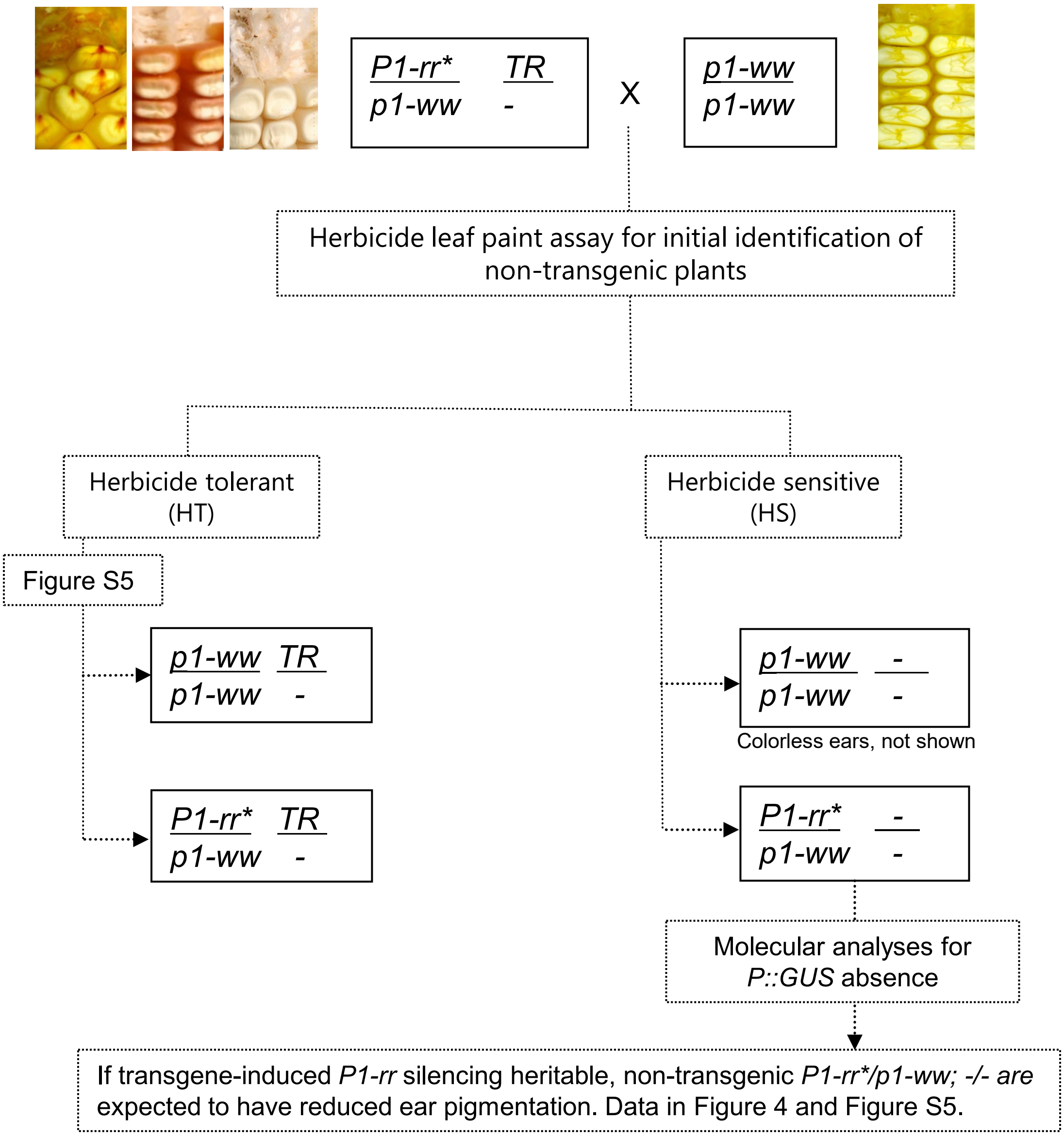


**Figure S4. Crossing scheme for testing heritability of transgene-induced *P1-rr* silencing in the absence of inducing transgene.** To test heritability of transgene induced *P1-rr* silencing, the progeny of transgenic plants that exhibited strong *P1-rr* silencing were analyzed. Representative ear phenotypes of the parental ears are shown on top. Initial identification of non-transgenic plants was by herbicide leaf paint assay (Materials and Methods). Because silencing of herbicide tolerance gene could occur, plants sensitive to herbicide in leaf paint assay were subjected to molecular analyses to confirm that they lacked a transgene (Materials and Methods). An asterisk (*) indicates an exposure of the *P1-rr* allele to a transgene.

| Construct | Event | # of plants | | Deviation from 1HT:1HS segreg. | # of *P1-rr**/ *P1-ww; -/-* plants |
|---|---|---|---|---|---|
| | | HT | HS | | |
| ***P1.2*** | P2P147-37 | 42 | 23 | | 15 |
| | P2P147-30 | 45 | 17 | | 10 |
| | P2P147-13 | 84 | 61 | | 34 |
| | P2P147-4 | 68 | 26 | | 17 |
| | P2P147-23 | 111 | 59 | ↑HT | 32 |
| | P2P147-9 | 48 | 30 | | 19 |
| | P2P147-28 | 54 | 49 | | 40 |
| ***Δ Δ 14+15*** | P2P104-44 | 84 | 50 | | 38 |
| ***13+15*** | P2P257-42 | 72 | 46 | | 26 |
| | P2P257-37 | 32 | 14 | | 14 |
| | P2P257-53 | 80 | 25 | ↑HT | 21 |
| | P2P257-36 | 51 | 25 | | 21 |
| | P2P257-21 | 28 | 27 | | 15 |
| | P2P257-33 | 27 | 17 | | 15 |
| | P2P257-55 | 44 | 27 | | 22 |
| ***15*** | P2P62-37 | 34 | 21 | | 8 |
| | P2P62-52 | 32 | 27 | | 15 |
| | P2P62-2 | 109 | 89 | | 45 |
| | P2P62-84 | 31 | 29 | | 16 |
| | P2P62-47 | 43 | 21 | | 11 |
| ***Δ 14+15α*** | P2P105-15 | 18 | 34 | | 17 |
| | P2P105-19 | 15 | 11 | | 10 |
| | P2P105-10 | 17 | 10 | | 6 |
| ***15β+Δ 13*** | P2P151-30 | 28 | 113 | ↓HT | 65 |
| | P2P151-20 | 42 | 41 | | 17 |
| ***15β*** | P2P152-12 | 23 | 25 | | 15 |
| | P2P152-22 | 43 | 26 | | 15 |
| | P2P152-1 | 34 | 23 | | 13 |
| | P2P152-9 | 152 | 66 | ↑HT | 34 |
| | P2P152-21 | 29 | 35 | | 20 |
| | P2P152-36 | 13 | 7 | | 1 |
| | P2P152-4 | 11 | 5 | | 4 |

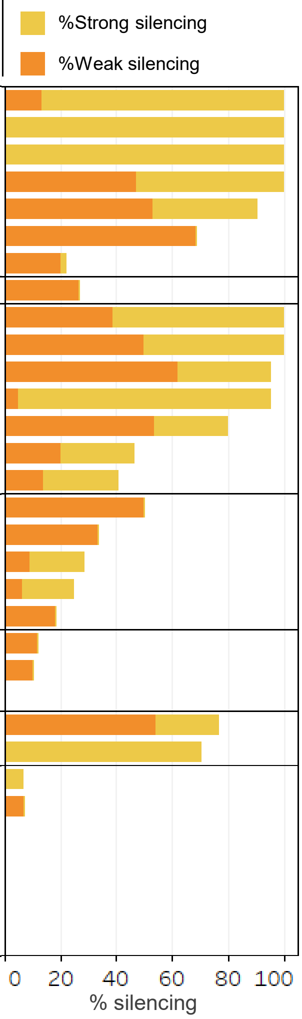


**Figure S5. Results of genetic test for heritability of transgene-induced *P1-rr* silencing in the absence of inducing transgene.** Construct and event names are shown in the first two columns. The third and fourth columns show number of herbicide tolerant (HT) and herbicide sensitive (HS) plants, respectively. Results of $\chi^2$ are shown in the fifth column. Events with greater than expected number of HT plants are indicated as HT↑, and events with lower-than-expected number of HT plants are indicated as HT↓. The null hypotheses was that HT and HS plants occurred at equal frequency. The null hypothesis was rejected when $\chi^2$ with one degree of freedom was greater than 3.84 (P = 0.05). The last column, shows number of the informative non transgenic *P1-rr**/*P1-ww; -/-* plants among HS plants. Absence of transgene in these plants was confirmed by molecular analyses. The bar chart on the right shows frequencies of weak (orange) and strong (yellow) transgene silencing for each event. To facilitate comparison between constructs, transgenic events within each construct were sorted from high to low frequency of silencing. Average frequency of silencing for each construct is also reported in Figure 4.

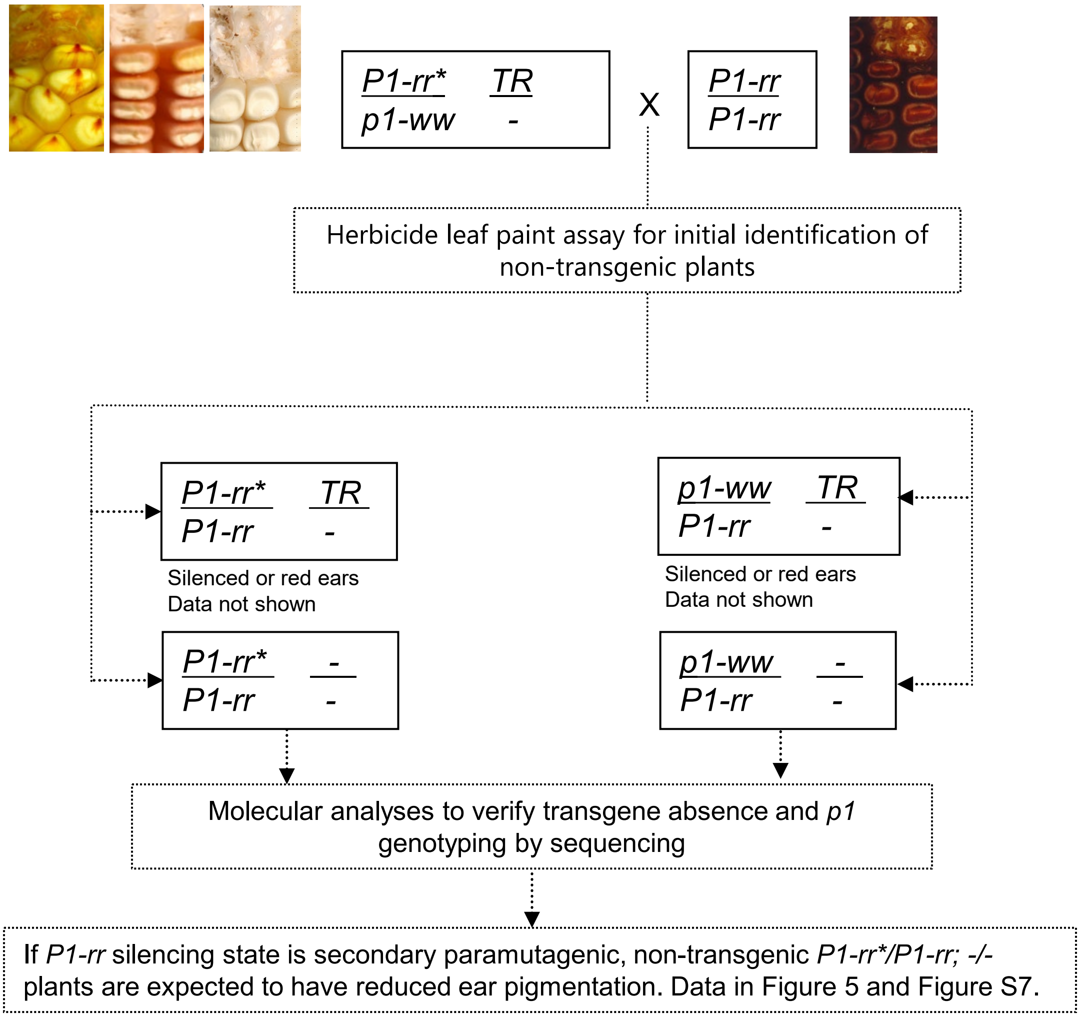


**Figure S6. Crossing scheme for testing secondary paramutagenicity of transgene-induced *P1-rr* silencing in the absence of an inducing transgene.** For a majority of transgenic events, *P1-rr*/p1-ww; TR/-* plants with strongly silenced ear phenotypes (representative ears shown on top) were crossed with *P1-rr.* Progeny plants were screened for herbicide tolerance by leaf paint assay. Plants that were sensitive to herbicide were confirmed to lack a transgene by DNA blot analyses. Genotyping of non-transgenic *P1-rr*/P1-rr; -/-* and *p1-ww/P1-rr; -/-* plants was by sequencing of PCR fragment spanning a polymorphism between the *P1-rr* and *p1-ww* alleles (Materials and Methods; location of polymorphism in Figure S1A). For two transgenic events of *P1.2* (indicated with superscript in Figure S7) starting material was homozygous for *P1-rr* (*P1-rr*/P1-rr*; TR/-*) and segregated only two progeny classes; *P1-rr*/P1-rr; TR/-* and *P1-rr*/P1-rr; -/-* (crossing scheme not diagrammed). An asterisk (*) indicates an exposure of the *P1-rr* allele to a transgene.

| Construct | Event | # of genotyped by sequencing non-transgenic plants | # of *p1-ww/ P1-rr; -/-* plants | # of *P1-rr*/ P1-rr; -/-* plants |
|---|---|---|---|---|
| ***P1.2*** | P2P147-9[1] | 24 | | 24 |
| | P2P147-4 | 21 | 9 | 12 |
| | P2P147-8[1] | 40 | | 40 |
| | P2P147-37 | 61 | 14 | 47 |
| ***ΔΔ14+15*** | P2P104-44 | 116 | 20 | 96 |
| ***13+15*** | P2P257-36 | 73 | 14 | 59 |
| | P2P257-42 | 160 | 68 | 92 |
| ***15*** | P2P62-84 | 28 | 12 | 16 |
| | P2P62-47 | 17 | 5 | 12 |
| | P2P62-52 | 47 | 15 | 32 |
| ***13+14+15α*** | P2P146-12 | 64 | 29 | 35 |
| ***Δ14+15α*** | P2P105-15 | 25 | 12 | 13 |
| ***15β+Δ13*** | P2P151-20 | 15 | 9 | 6 |
| | P2P151-30 | 10 | 4 | 6 |
| | P2P151-32 | 37 | 13 | 24 |
| ***15β*** | P2P152-22 | 92 | 47 | 45 |

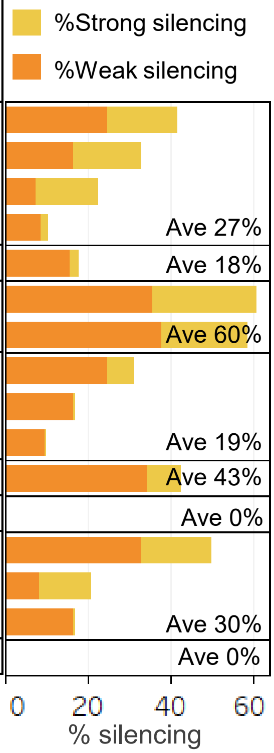


**Figure S7. Results of genetic test for secondary paramutation of transgene-induced silencing of *P1-rr*.** Crossing scheme is diagrammed in Figure S6. the first two columns show construct and event names. The third column shows number of non-transgenic plants genotyped by sequencing (Materials and Methods). The fourth and the fifth columns show the number of non-transgenic plants with *p1-ww/P1-rr;-/-* and *P1-rr*/P1-rr;-/-* genotypes, respectively. Chart shows frequencies of weak (orange) and strong (yellow) silencing among the informative *P1-rr*/P1-rr;-/-* progeny of each tested event. Average frequency of silencing for each construct is also shown for convenience (also in Figure 5). To improve comprehension of the results, transgenic events within each construct were sorted from high to low frequency of *P1-rr* silencing.

[1] For two transgenic events of *P1.2* starting material was homozygous for *P1-rr* (*P1-rr*/P1-rr*; TR/-*) (not diagrammed in Figure S6). Thus, progeny of these crosses contained no *p1-ww/P1-rr* plants.

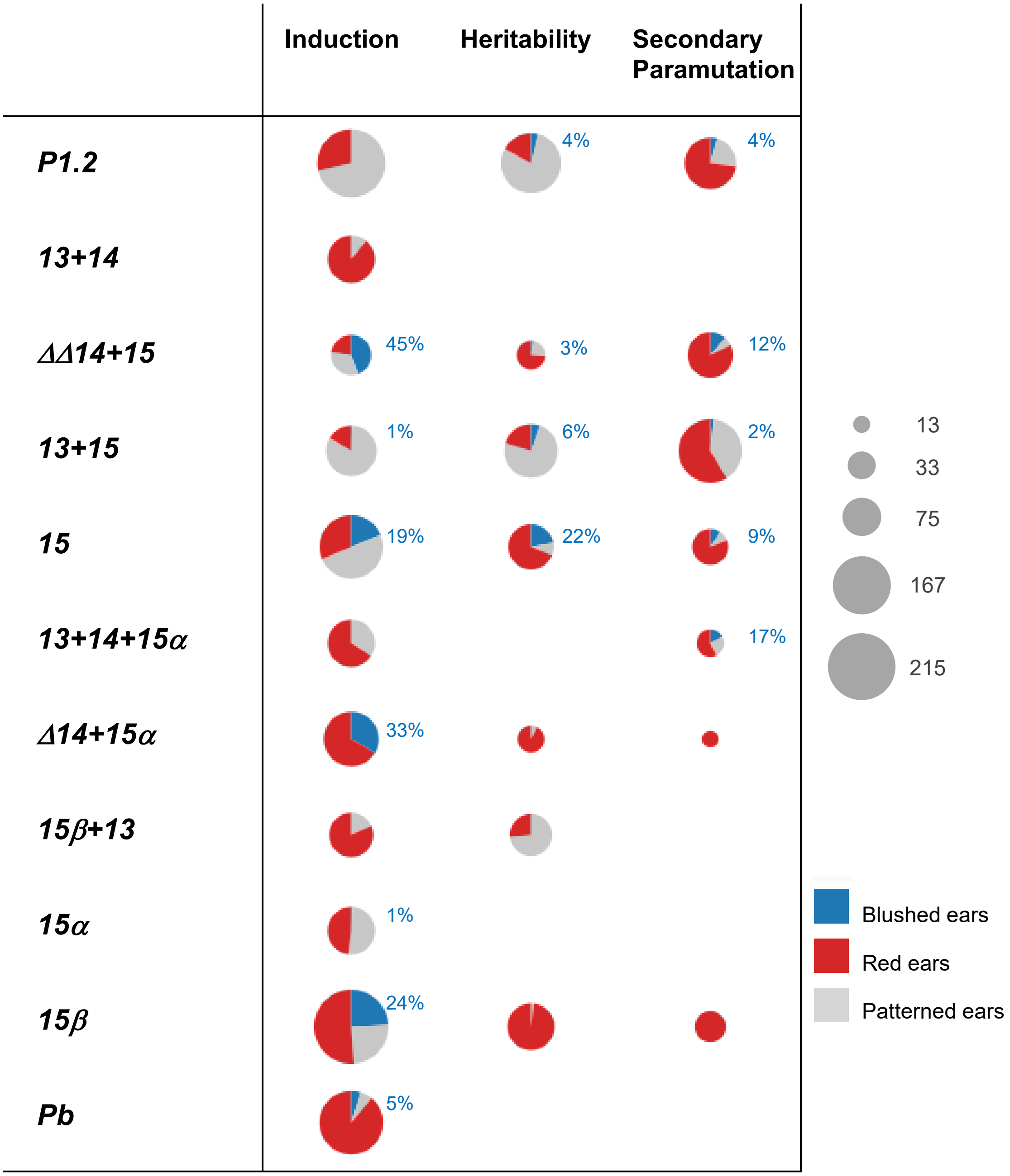


**Figure S8. Frequency of patterned and blushed *P1-rr'* ear phenotypes in genetic tests.** Genetic tests (induction of silencing, heritability, and secondary paramutation) are indicated on the top. Pie sizes are proportional to the number of informative plants tested (as detailed in Figures 3, 4, and 5). Percent of blushed ears is indicated in blue.

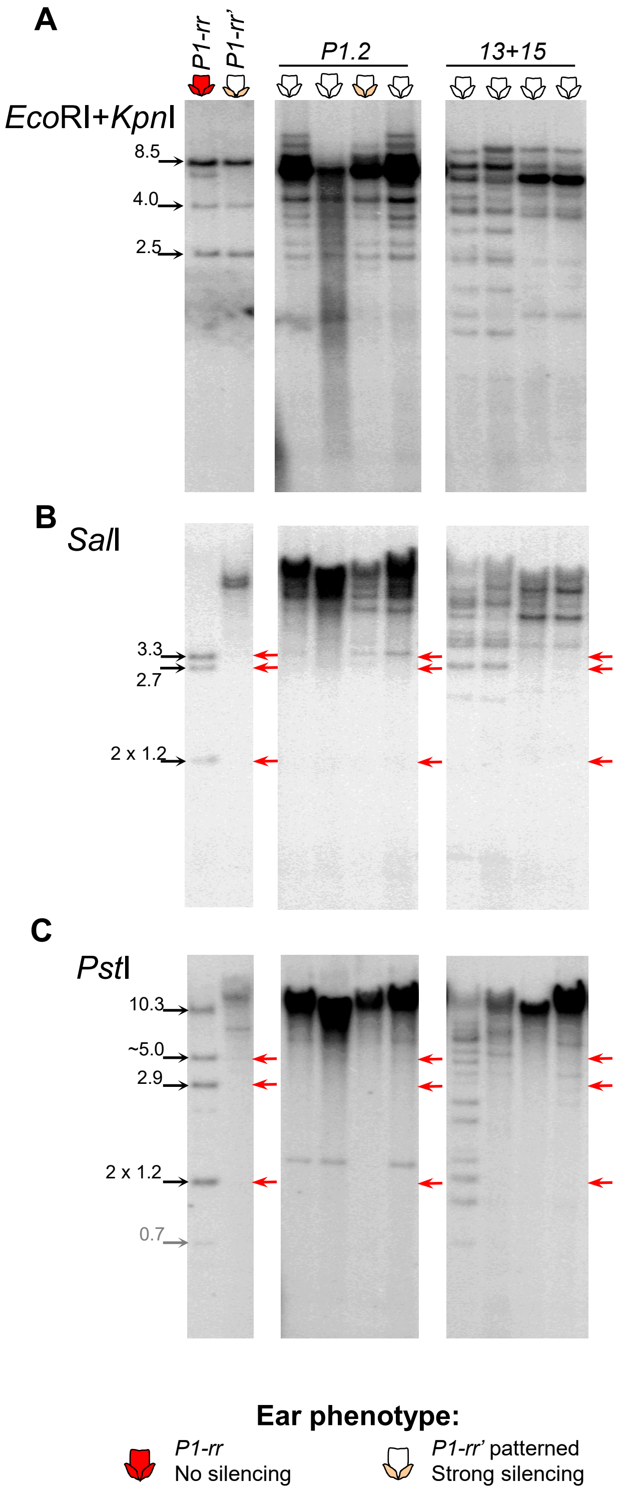
A
P1-rr
P1-rr'
P1.2
13+15
EcoRI+KpnI
8.5
4.0
2.5
B
SalI
3.3
2.7
2 x 1.2
C
PstI
10.3
~5.0
2.9
2 x 1.2
0.7
Ear phenotype:
P1-rr
No silencing
P1-rr' patterned
Strong silencing

# Figure S9

**Figure S9. DNA blot analysis of transgenic plants carrying *P1.2* and *13+15* transgenes.** In panels **A** through **C**, leaf genomic DNA was hybridized with the $^{32}P$ labeled probe 15. Assayed *P1.2* transgenic plants were from the P2P147-6 event. For *13+15,* two events were assayed: P2P257-36 (first and second plant on *13+15* blots) and P2P257-33 (third and fourth plants on *13+15* blots). Pigmentation of kernels and cob glumes of the assayed plants are shown as pictograms above each lane. Lanes for *P1-rr* and *P1-rr'* controls are duplicated with Figure S11; *P1.2* event P2P147-6 (this figure) and *15β* event P2P152-12 (Figure S11) were analyzed on the same DNA blot and shared the controls. Black arrows on the left of the blots indicate fragment sizes in kilobases (kb), while red arrows on the right of the blots indicate expected location of bands that shifted up as a result of cytosine methylation within *Sal*I and *Pst*I restriction sites. Bands that do not align with expected location of the endogenous *P1-rr* bands are presumed to be transgenic. **A.** Blots for *Eco*RI+*Kpn*I digestion. *Eco*RI and *KpnI* are not sensitive to cytosine methylation. Digestion of *P1-rr* and *P1-rr'* genomic DNA results in ~8.5, 4.0 and 2.5 kb bands. **B.** Blots for *Sal*I digestion. *Sal*I is sensitive to cytosine CpG methylation. Digestion of *P1-rr* DNA with *Sal*I results in 3.3, 2.7 and 1.2 (doublet) kb bands. Digestion of *P1-rr'* results in high molecular weight bands >10 kb. **C.** Blots for *Pst*I digestion. *Pst*I is sensitive to CpHpG cytosine methylation (where H is any base but G). Restriction digestion of *P1-rr* DNA results in 10.3, ~5.0, 2.9 and 1.2 (doublet) kb bands. A faint 0.7 kb band (indicated in gray font) is detected in *P1-rr.* Weak signal from this band prevented reliable assessment of this band presence/absence in transgenic samples. Digestion of *P1-rr'* DNA results in high molecular weight bands (>10 kb).

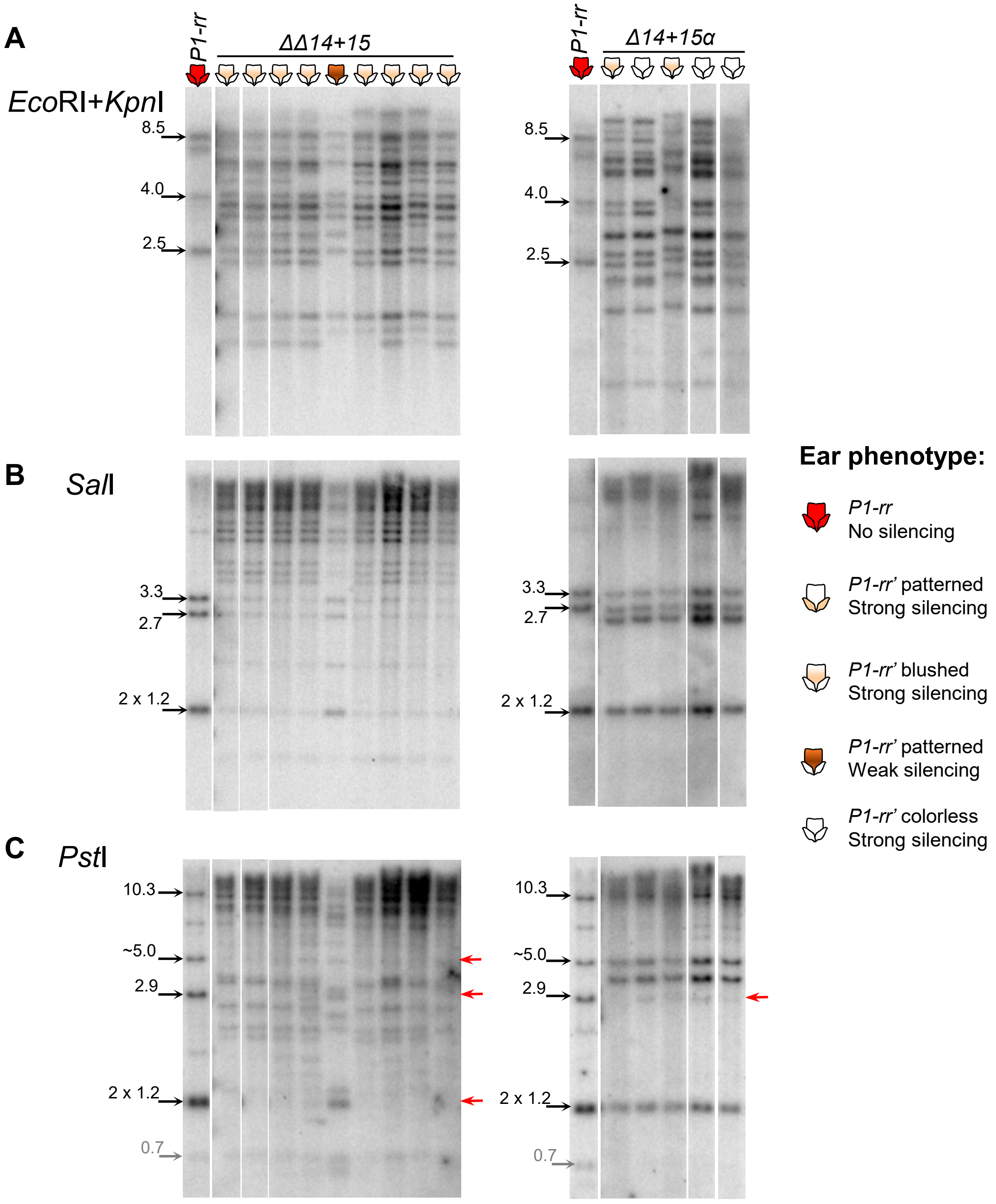
A
EcoRI+KpnI
P1-rr
ΔΔ14+15
Δ14+15α
8.5
4.0
2.5
B
SalI
3.3
2.7
2 x 1.2
C
PstI
10.3
~5.0
2.9
2 x 1.2
0.7
Ear phenotype:
P1-rr
No silencing
P1-rr' patterned
Strong silencing
P1-rr' blushed
Strong silencing
P1-rr' patterned
Weak silencing
P1-rr' colorless
Strong silencing

# Figure S10

**Figure S10. DNA blot analysis of transgenic plants carrying the *ΔΔ14+15* and *Δ14+15α* sub-fragments of *P1.2*.** In panels **A** through **C**, leaf genomic DNA was digested with restriction enzymes and hybridized with the $^{32}$P labeled probe 15. Transgenic plants assayed were from the following events: the P2P104-44 event for *ΔΔ14+15* and the P2P105-1 event for *Δ14+15α.* Ear phenotypes of transgenic plants are shown as pictograms above each lane. Black arrows on the left of the blots indicate fragment sizes in kilobases (kb), while red arrows on the right of the blots indicate expected location of bands that shifted up as a result of cytosine methylation within *Sal*I and *Pst*I recognition sites. Bands that do not align with expected location of the endogenous *P1-rr* bands are presumed to be of transgenic origin. **A.** Blots for *Eco*RI+*Kpn*I digestion. *Eco*RI and *KpnI* are not sensitive to cytosine methylation. Digestion of *P1-rr* genomic DNA results in ~8.5, 4.0 and 2.5 kb bands. **B.** Blots for *Sal*I digestion. *Sal*I is sensitive to cytosine CpG methylation. Digestion of *P1-rr* DNA with *Sal*I results in 3.3, 2.7 and 1.2 (doublet) kb bands. **C.** Blots for *Pst*I digestion. *Pst*I is sensitive to CpHpG cytosine methylation (where H is any base but G). Restriction digestion of *P1-rr* DNA results in 10.3, ~5.0, 2.9 and 1.2 (doublet) kb bands. A faint 0.7 kb band, indicated in gray, is also detected in *P1-rr.* Weak signal from this band prevented reliable assessment of this band presence/absence in transgenic samples.

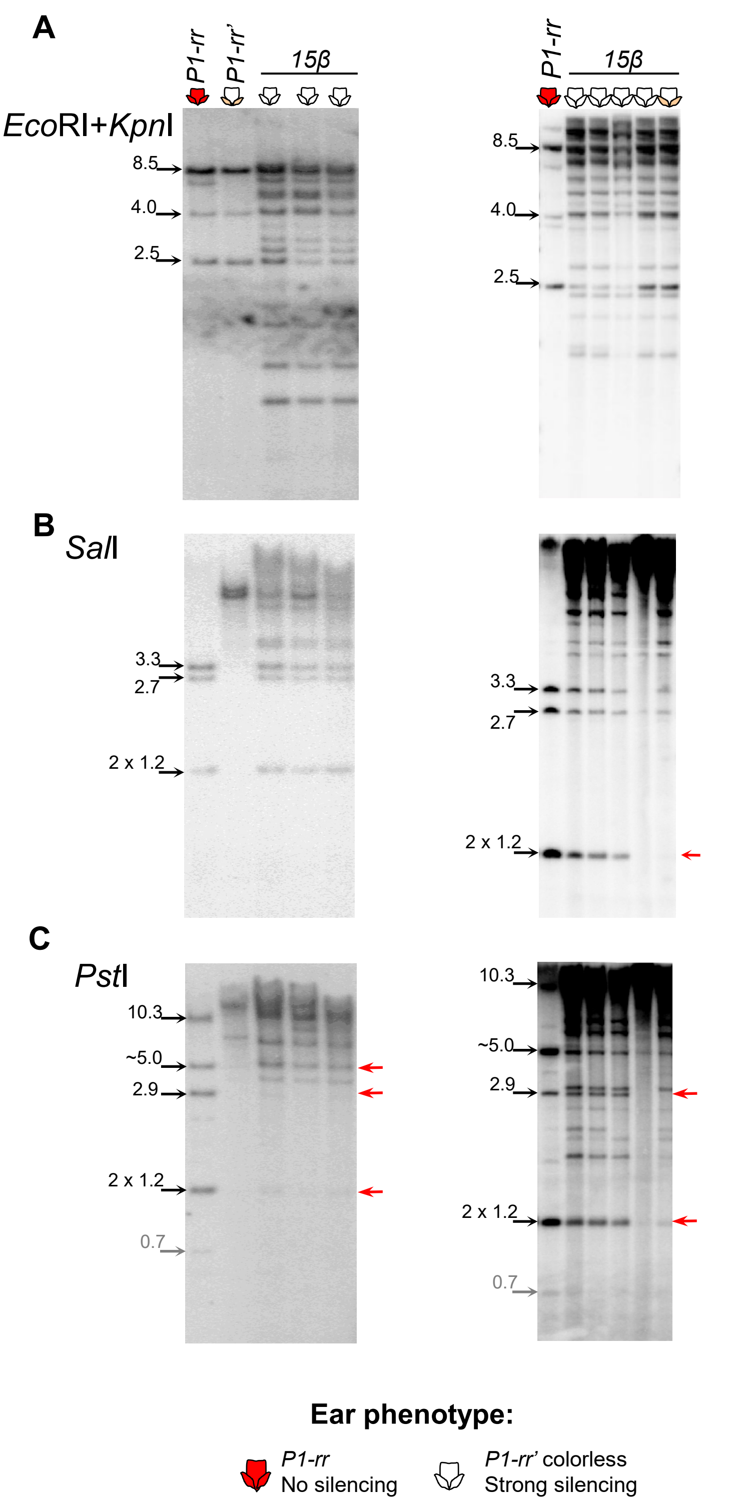

A
P1-rr
P1-rr'
15β
EcoRI+KpnI
8.5
4.0
2.5
B
SalI
3.3
2.7
2 x 1.2
C
PstI
10.3
~5.0
2.9
2 x 1.2
0.7
Ear phenotype:
P1-rr
No silencing
P1-rr' colorless
Strong silencing


# Figure S11

**Figure S11. DNA blot analysis of transgenic plants carrying *15β* transgenes.** In panels **A** through **C**, leaf genomic DNA was hybridized with the $^{32}P$ labeled probe 15. Transgenic plants were from *15β* events P2P152-12 (left) and P2P152-36 (right). Lanes for *P1-rr* and *P1-rr'* controls are duplicated with Figure S9; *P1.2* event P2P147-6 and *15β* event P2P152-12 were analyzed on the same DNA blot and, therefore, share the controls. Pigmentation of kernels and cob glumes of the assayed plants are shown as pictograms above each lane. Black arrows on the left of the blots indicate fragment sizes in kilobases (kb), while red arrows on the right of the blots indicate expected location of bands that shifted up as a result of cytosine methylation within *Sal*I and *Pst*I recognition sites. Bands that do not align with expected location of the endogenous *P1-rr* bands are presumed to be of transgenic origin. **A.** Blots for *Eco*RI+*Kpn*I digestion. *Eco*RI and *KpnI* are not sensitive to cytosine methylation. Digestion of *P1-rr* and *P1-rr'* genomic DNA results in ~8.5, 4.0 and 2.5 kb bands. **B.** Blots for *Sal*I digestion. *Sal*I is sensitive to cytosine CpG methylation. Digestion of *P1-rr* DNA with *Sal*I results in 3.3, 2.7 and 1.2 (doublet) kb bands. Digestion of *P1-rr'* results in high molecular weight bands >10 kb. **C.** Blots for *Pst*I digestion. *Pst*I is sensitive to CpHpG cytosine methylation (where H is any base but G*). Pst*I digestion of *P1-rr* DNA results in 10.3, ~5.0, 2.9 and 1.2 (doublet) kb bands. A faint 0.7 kb band, indicated in gray, is also detected in *P1-rr.* Weak signal from this band prevented reliable assessment of this band presence/absence in transgenic samples. Digestion of *P1-rr'* DNA results in high molecular weight bands (>10 kb).
