# Supplementary material for: Transcribed enhancer sequences are required for maize p1 paramutation
Source: Genetics. 2024 Jan 3;226(1):iyad178. doi: 10.1093/genetics/iyad178 (PMC10763531; doi:10.1093/genetics/iyad178)
Supplement: iyad178_Supplementary_Data [file iyad178_supplementary_data.zip › Figure_S1_GENETICS-2023-306435.pdf]

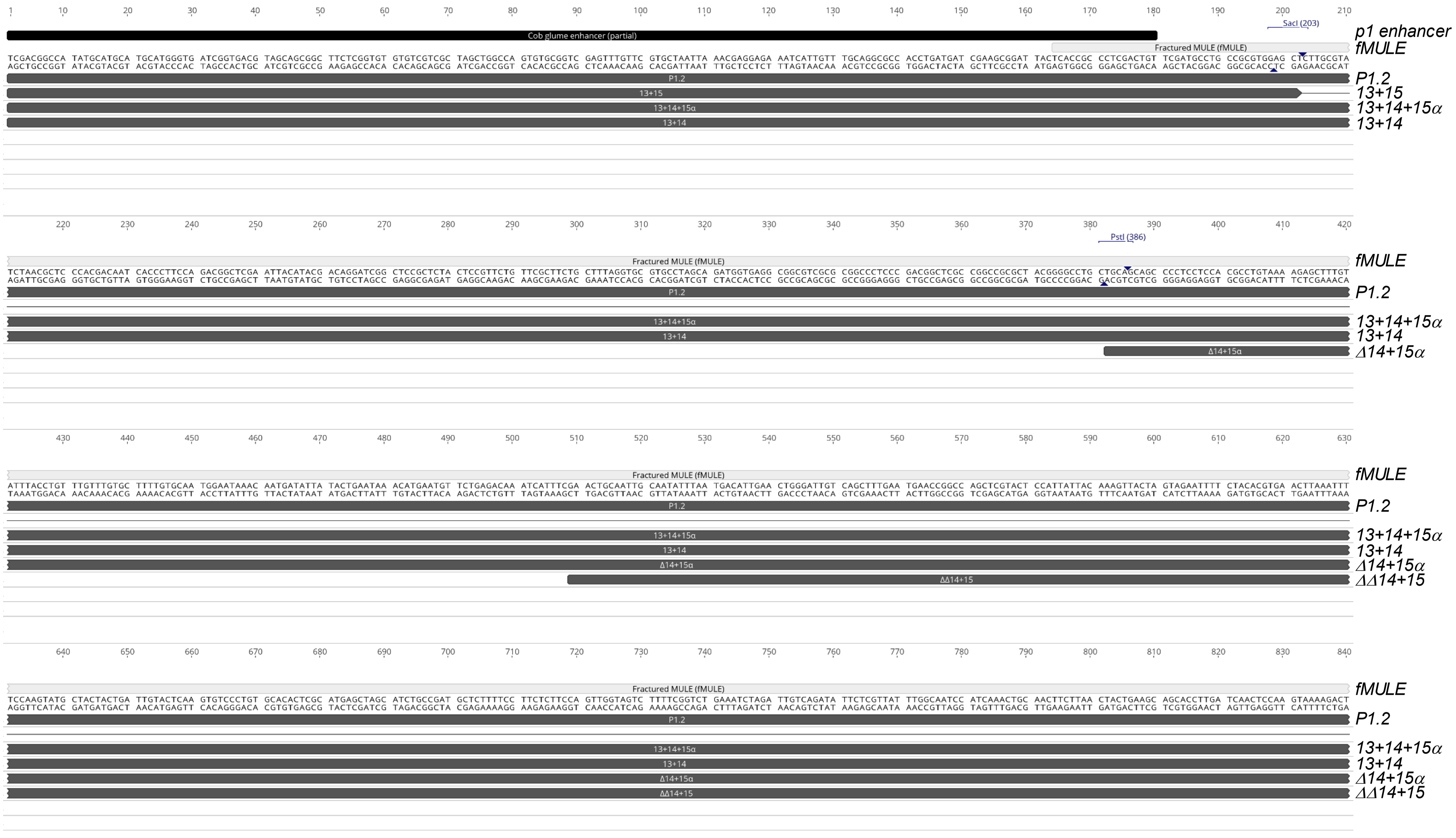

Figure S1 A, page 1

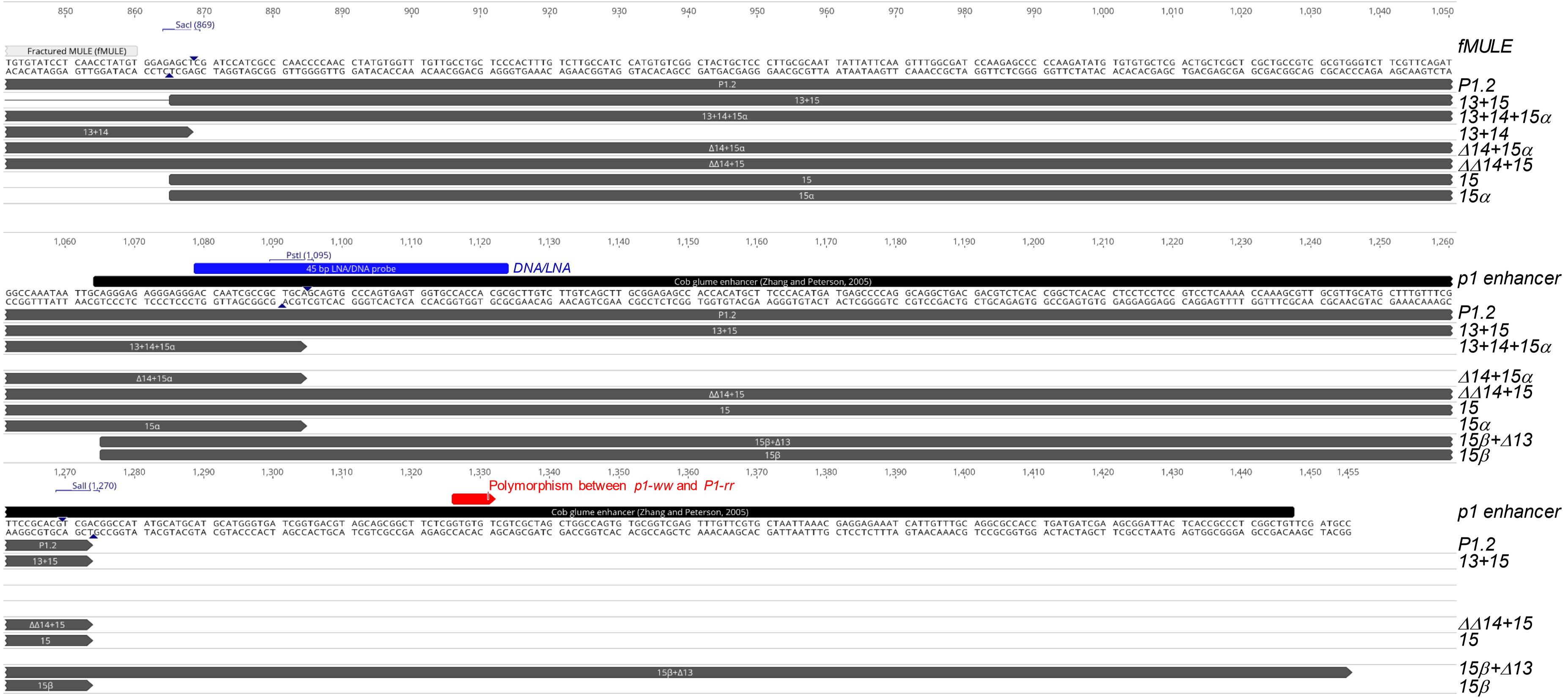

Figure S1 A, page 2

B

Probe 13	TCGACGGCCA	TATGCATGCA	TGCATGGGTG	ATCGGTGACG
13	TCGACGGCCA	TATGCATGCA	TGCATGGGTG	ATCGGTGACG
Δ13	TCGACGGCCA	TATGCATGCA	TGCATGGGTG	ATCGGTGACG
Probe 13	TAGCAGCGGC	TTCTCGGTGT	GTGTCGTCGC	TAGCTGGCCA
13	TAGCAGCGGC	TTCTCGGTGT	GTGTCGTCGC	TAGCTGGCCA
Δ13	TAGCAGCGGC	TTCTCGGTGT	GT--CGTCGC	TAGCTGGCCA
Probe 13	GTGTGCGGTC	GAGTTTGTTC	GTGCTAATTA	AACGAGGAGA
13	GTGTGCGGTC	GAGTTTGTTC	GTGCTAATTA	AACGAGGAGA
Δ13	GTGTGCGGTC	GAGTTTGTTC	GTGCTAATTA	AACGAGGAGA
Probe 13	AATCATTGTT	TGCAGGCGCC	ACCTGATGAT	CGAAGCGGAT
13	AATCATTGTT	TGCAGGCGCC	ACCTGATGAT	CGAAGCGGAT
Δ13	AATCATTGTT	TGCAGGCGCC	ACCTGATGAT	CGAAGCGGAT
Probe 13	TACTCACCGC	CCTCGACTGT	TCGATGCCTG	CCGCGTGGAG
13	TACTCACCGC	CCTCGACTGT	TCGATGCCTG	CCGCGTGGAG
Δ13	TACTCACCGC	CCTCGGCTGT	TCGATGCC	
Probe 13	CTC			
13	CTC			

Figure S1. Sequence of the *P1.2* fragment and its sub-fragments. A. Sequence of the *P1.2* fragment continuous with the downstream copy of the *Δ13* sub-fragment. Locations of *p1* cob glume enhancer (Zhang and Peterson, 2005; Zhang and Peterson, 2006) and 45 bp DNA/LNA probes (Figure 7) are shown in black and blue, respectively, above the DNA sequence. Transgenic sub-fragments are diagrammed in dark gray below the DNA sequence. Polymorphism between *p1-ww* and *P1-rr* that was used for genotyping in secondary paramutation test (Materials and Methods) is shown in red. **B.** Alignment of *P1-rr* probe 13 (Lechelt *et al.,* 1989) and the *P1.2* sub-fragments *13* and *Δ13.*
